# Observing teaching principles in school - Development and Validation of an Observation Protocol (OPTIS)

**DOI:** 10.1371/journal.pone.0355710

**Published:** 2026-08-17

**Authors:** Theres Mühlberg, Joachim Bachner, Filip Mess, Jan Schmid-Ellinger

**Affiliations:** Technical University of Munich, TUM School of Medicine and Health, Department of Sport and Health Sciences, Associate Professorship of Didactics in Sport and Health, Am Olympiacampus, Munich, Germany; GrandEdu GmbH, GERMANY

## Abstract

Instructional quality seems to have a considerable impact on students’ achievements. Unpacking instructional quality through observation is an increasingly used method in educational research. Although a great variety of observational measurements for school observation are available, there is a lack of a valid, reliable, and subject-independent observation protocols. Furthermore, observation protocols that do not solely serve evaluation purposes, and are based on a constructivist learning tradition and a comparative literature analysis of existing teaching principles are missing. This study aims to address this lack by developing an observation protocol that helps observers to identify the applied teaching principles during class. The developmental validation process of the observation protocol *Observing Principles of Teaching In School (OPTIS)* is detailed, and the piloting via video-recorded and real-time teaching is described. Inter- and intra-observer reliability, computed with Gwet’s AC2, for two independent observers achieved an almost perfect agreement. Several expert rounds and theoretical foundations provided evidence for high predictive validity. Overall, test- and piloting results present evidence regarding the psychometric properties of the OPTIS protocol.

## Introduction

Results from the Programme for International Student Assessment (PISA) in 2022 expose that students’ competencies regarding mathematics, science, and reading across countries of the Organization for Economic Co-operation and Development (OECD) are decreasing [1]. For years, education policy has been striving to identify points of departure and causes to enhance educational quality (e.g., instructional quality, educational equity, etc.) based on student achievement results. Already back in 2012, the OECD countries articulated one goal: improving instructional quality with the help of evaluation of schools and teaching [2]. Despite the variety of reform initiatives implemented to enhance instructional quality, the effectiveness of these endeavors has only been evaluated using student outcome scores on standardized achievement tests. Until today, these tests have mainly aimed at comparing student achievements (e.g., across countries) to derive reforms focusing on the identified desiderata. The evaluation of these reforms, again, is usually conducted solely based on student achievement tests. Within this cycle, test scores in large-scale education studies measuring students’ achievements can neither explain how nor why students produce these results. Another approach could focus on the process of teaching and learning, rather than solely focusing on the students’ outcomes (the product). This approach could lead, for example, to monitor the quality of instruction, which is widely regarded as the most influential factor in explaining a considerable portion of the variance in student outcomes [3–7]. In addition, analyzing instructional quality, by focusing on the process of teaching and learning with the help of valid and reliable observation protocols, could help identify strengths and weaknesses during teaching, which may later help to improve current teaching practices [8,9]. The dissemination of this approach might have received little attention so far due to the unclear concept of instructional quality and suitable observation instruments in research and practice. Therefore, first, it is necessary to clarify the concept of instructional quality before presenting the development of an observation protocol, named OPTIS.

## Instructional quality within a constructivist understanding of learning

To discuss key aspects of instructional quality, educational research in recent years has increasingly addressed the questions: What is *high-quality instruction*? And what is *good teaching*? Considerable progress has been made in conceptualizing, operationalizing, and measuring instructional quality [10]. Various frameworks and models have emerged to clarify their implication (e.g., [4,11]), yet approaches differ widely depending on research traditions and often use inconsistent terminology. Most often, instructional quality research investigates *what* works in education and *why* [12]. Currently, this research is mainly connected with educational effectiveness research [EER; 13–15] and therefore to the question ‘What are effective teaching practices?’ [3,16,17]. This effectiveness is most often measured with students’ outcomes, (e.g., grades/PISA) but leaves the progress out of consideration. There is ongoing criticism in research that the discussion of instructional quality is aligned with effectiveness research and students’ achievements, respectively [18–20]. In EER, however, instructional quality is considered one of the major modifiable factors influencing student outcomes [21,22]. This perspective highlights the importance of considering learning and teaching processes, which is in the center of attention when discussing instructional quality.

### Dimensions of instructional quality

Instructional quality is often described with the help of different dimensions. This approach can be traced back to Walberg [23] and Carroll [24]. Since then, almost countless collections of dimensions of instructional quality have been published [4,25]. In German-speaking countries, the Three Basic Dimensions Framework, with its ‘MAIN-Teach-Modell’, is one of the few systematically disseminated frameworks of generic dimensions of instructional quality [26–28]. Other widespread terminologies are *dimensions of teaching qualit*y [29], *teaching principles* [10,30,31], *sub-dimensions of instructional quality* [11,26] or *elements of good teaching* [15]. This represents a small insight into the inconsistent use of wordings like dimensions, elements or principles. For this paper, as outlined by Klieme et al. [27], we refer to dimensions of instructional quality as overarching categories. As introduced by Helmke [10] or Wiater [30], these dimensions in turn contain sub-dimensions, which are referred to as *teaching principles*. These teaching principles are considered general principles for successful teaching or principles which design learning and teaching processes [32].

As Schlesinger and Jentsch [11] and already Einsiedler [33] pointed out, there remains inconsistency in research regarding the most pertinent generic teaching principles. Collections of generic teaching principles are provided by, for example, Helmke [10], Brophy [15], Hattie [22], Wiater [30], Kiel [31], Kunter [34], Clausen [35], Meyer [36]. Most of these collections are primarily based on empirical research, rather than on theoretical foundations or learning theories [11,14,26,37,38]. Different collections of teaching principles differ with respect to certain characteristics, such as educational level, intended purpose or subject relatedness [5,19,21,39–41]*.* Following general educational research, teaching principles are to be discussed without favoring one specific subject and should be applicable to teaching in general [30,34,41].

At the same time, however, discussing instructional quality is always linked to a specific perspective on school and teaching [42]. Well-known perspectives on learning and teaching are provided by, for example, behaviorism, cognitive learning theory, social learning theory, situated learning theory or constructive learning theory [43]. In line with the outlined understanding of teaching principles, a strong emphasis is placed on the process of designing teaching and learning, which aligns with the constructivist approach of learning. This is one of the main reasons why the constructivist learning theory forms the basis of several collections of teaching principles [10,15,31,44]. Originating in the 1990s, the *constructive learning theory* [45,46] became popular and has not lost its popularity until today [47–49]. According to this constructivist understanding, learning is a process in which learners actively build up their knowledge by interfering with other subjects and people in diverse learning environments. The constructivist learning theory describes learning in various contexts, not only schools. Therefore, the term ‘learners’ is used in this section. Following constructivism, learners are able to actively construct their knowledge through examples and subsequently apply it in various contexts. This can differ due to individual pre-knowledge and individual constructive processes from learners [49–51]. Furthermore, learning needs authentic surroundings and problems to constructively provoke learning [52–54].

With regard to constructivism, some criteria of instructional quality have been rarely represented by existing collection of teaching principles and thus should be additionally considered. Those criteria could be, for example, to *create new experiences* so learners can transfer their own knowledge [55]. Or a *connection to pre-knowledge* is essential to build on it, and *authentic learning environments* are needed to implement theoretical knowledge of real-world phenomena [49,52]. Besides this, creating learning content and spaces should be a *participative process* in which learners’ interests and needs are taken into account [56]. Additionally, *learning environments* should be brought more into focus, and *cross-disciplinary* teaching can help to transfer knowledge between different fields because learning is regarded as a flexible process in a constructivist understanding [50,57].

However, what prompts the necessity to enumerate all kinds of teaching principles? Within educational research, these teaching principles are used to analyze and enhance teaching [38]. Applying these principles to measure current teaching practice can connect research with practice and assist in assessing the strengths and weaknesses of teaching, thereby measuring current instructional quality. Furthermore, observing teaching principles during teaching can put the focus on monitoring current teaching processes as a highly modifiable factor in the classroom.

## Observational measurements in school

Observation in general is a social science method for data collection in which the issue of interest is observed and “relevant facts, actions and behaviors” are captured [58]. Observing is considered as one of the most common and objective measurements for identifying and analyzing teaching [11,21,59,60]. In general, school observations are often used by principals for formative feedback to teachers, for teachers’ performance evaluation, or as part of professional teachers’ development programs [4,61,62]. In research, school observation is frequently employed to assess teachers’ performances in correlation to students’ learning outcomes [14,37,63]. Those observations are often supported by descriptive or rating-based observation protocols to generate meaningful data. The necessity for these observation-supporting measurements was investigated by Strong et al. [64]. They showed that less than 50% of observers could identify ‘effective’ teaching without using supporting measurements. Up to today, a great variety of observational measurements have been developed, which differ enormously in their design and application (c.f. Chapter ‘methods‘). Ladics et al. [63] reviewed 39 existing observation protocols from 30 countries. They indicated that throughout all reviewed observation measurements, only very few specifically measure classroom practice in a reliable manner. Resulting from these findings, researchers demand reliable standards for developing observational measurements and call for well-validated protocols to minimize validity problems of behavioral observation [65].

To the knowledge of the authors of this paper, only three existing structured observation protocols meet these needs to some extent and provide generic, subject-independent information with acceptable reliability: TEACH [4], “Einblicknahmen in die Lehr-Lernsituation” (ELL, [66]) and “Unterrichtsbeobachtungsbogen” (UBB, [48]). TEACH was specifically developed for low- and middle-income countries as a result of bad circumstances in classrooms and unsatisfactory student outcome results. ELL is exclusively based on Helmke’s theory and collection of teaching principles (which are based on an empirical derivation of instructional quality). The UBB, comprising five pages, appears to be too long for a straightforward, practical application and was created for learners from nursery to elementary school levels. All three observation protocols lack a consistent theoretical foundation, such as an explicit grounding in constructivist learning theory or a comprehensive comparative review of the literature. Incorporating such foundations would strengthen these instruments by situating them more clearly within the educational research field.

In summary, classroom observation is regarded as a useful, meaningful and objective measurement for analyzing teaching [18,59,60,65]. Nevertheless, there are still some limitations to current observational practice and available observation protocols. Firstly, existing observation protocols are rarely based on collaborative work or built upon a theoretical foundation. As detailed above, when observation protocols are based on teaching principles, these are mostly exclusively drawn from empirical research, which could lead to important aspects or principles being disregarded. Secondly, a lot of available observation protocols do not measure generic dimensions of teaching, but assess teaching as either subject- [67–69] or topic-specific [59,65]. Thirdly, existing observation protocols most often serve teacher evaluation purposes and do not focus on analyzing current teaching practice, as intended in the presented understanding of monitoring instructional quality. As indicated by Praetorius and Charalambous [26], it is essential to define the specific intended use of an observation protocol in order to evaluate the validity of its measurement. However, this clarity is often lacking in current practice. Fourthly, available observation protocols are rarely checked for reliability [65,70] and if so, the reliability process needs to be described better and more consistently [21]. Fifthly, many observation protocols seem unsuitable for real-time observation because of the large extent they cover [48].

Therefore, the aim was to develop an observation protocol that accounts for those desiderata. By working collaboratively, as requested by Praetorius and Charalambous [26], the observation protocol *Observing Principles of Teaching In School* (OPTIS) was developed by combining empirical and theoretical research, a learning theory, empirical data and experts’ feedback. In the following section, the development and validation processes are documented in detail.

## Methodology and instrument development

**Fig 1:**
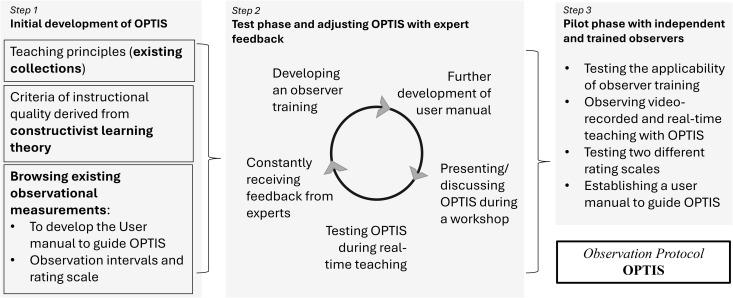
Developmental process for OPTIS.

The development of OPTIS underwent multiple steps and took 15 months in total. The ethics committee of the Technical University of Munich (Institute of Medicine), Germany approved this study (Approval code 2022–464-S-SR). The initial list of teaching principles, on which OPTIS is based on, was drawn from existing collections of teaching principles. These teaching principles were supplemented with additional quality criteria of instructional quality, which was needed to ground OPTIS in the constructivist learning theory (see Fig 1, first step).

Afterwards experts from school, educational science, observation practice, and university reviewed different stages of the development of the observation protocol and helped adjust and clarify each item. Overall, ten experts provided feedback during the developing process, and additional nine experts discussed the items of the observation protocol within a workshop (see Fig 1, second step). One test phase and a final piloting phase completed the overall developmental process (see Fig 1, third step). Furthermore, inter- and intra-observer reliability was computed based on video-recorded (n = 9) teaching and field data (n = 13). The whole development process is presented in the following. At the end of this section, the final version of OPTIS is displayed (Table 1), and the complete observation protocol is available in the appendix (S2).

**Table 1:** The teaching principles and their observation categories OPTIS consists of when going into its final piloting.

	Teaching principles	Observation categories
**(Cognitive) activation**	**Action orientation**	Content is developed by the students through their own actions (these actions serve as a starting point for further teaching activities)
	**Experience-based**	Students can gain their own experience in the classroom
	**Holistic approach**	Different senses of the students are addressed in class (learning with head, heart & hand)
	**Competence orientation**	The significance of the subject matter is made clear to the students by the learning objectives (present significance/future significance)
		Students assess their performance and competencies themselves
	**Motivation**	The students learning & performance needs are taken into account, awakened & maintained
	**Target orientation**	Learning objectives are clearly formulated by the instructor & made clear to the students
**Lesson design**	**Process orientation**	The learning paths/the learning process is perceived as an important sub-component by the teacher
	**Independence**	Independent learning is encouraged and challenged in a variety of ways in the classroom
	**Learner orientation**	The teacher aligns the lessons to the interests and needs of the students
	**Participation**	Students are given space to help shape and design the lesson
	**Experiencing self-efficacy**	The lessons open up for individual learning and solutions
	**Cooperative learning**	Students support each other in their learning/ social interactions take place
	**Variety of methods**	The teacher uses various methods to organize the lessons
	**Visualization**	The learning processes are supported by the use of various media by the teacher
	**Transparent performance expectations**	The instructor makes the requirements transparent to the students/ The students know what is expected from them at all times
	**Intelligent practice**	The exercise phases fit in with the lessons
	**Fuse of content**	What has been learned is practiced, repeated, deepened and secured sufficiently often by the students
	**Differentiation**	The LK supports different learning speeds, learning paths & performance levels
		Students receive differentiated feedback and assistance
**Contents & Topics**	**Activate prior knowledge**	The content can be linked to previous knowledge and experience
	**Interdisciplinary**	There is a transfer between different subjects
	**Authentic & problem-based learning experiences**	The lessons contain examples from everyday life/ from the students’ living environment/ address problems
**Classroom management**	**Structure & clarity**	The lessons have a common thread (lesson content builds on each other)
		The teaching process is based on recognizable rules
	**Time utilization** (High proportion of real learning time)	Answers of the students or work phases are not interrupted by the teacher
		Students are busy with learning activities/lesson-related content almost all the time
	**Communication**	The volume is appropriate to the social form & learning content
		The teacher works with non-verbal communication (gestures/mimic/rituals)
		Work assignments, explanations & impulses are formulated clearly, precisely & coherently
**Constructive** **support**	**Learning-friendly** **classroom climate**	The teacher recognizes and encourages the students’ willingness to learn
		Dealing with mistakes in a way that promotes learning/ students’ contributions are seen as learning opportunities
		The teacher encourages the students to ask questions/express their thoughts
		There is an atmosphere of mutual appreciation and respect between teachers and students
		…. and between students and students
**Learning space**	**Learning environment**	The choice of learning environment matches with the teaching topic
		The learning environment matches the learning phase
		Students explore the learning environment through movement and interaction
	**Use of the learning environment**	The learning environment is actively integrated into the lesson (it becomes the point of departure of learning)

### Development of OPTIS

#### Step 1 – Initial development of OPTIS based on exiting collection of teaching principles, the constructivist learning theory and existing observation protocols.

When developing an observation protocol, there are different developmental approaches in research [26]. Either, researchers came up with a first catalog of observation items gathered from literature [71–76], they built their protocols on existing observation protocols [11,59,61,77,78] or based their protocols on a mixture of both approaches [4,48]. These approaches were followed either by a group of experts reviewing and discussing the initial selection of observation items [70–72,75,77], by piloting the protocol directly in the field [59,74] or by adjusting the initial items based on the observation of video-recorded teaching [4]. Each of these approaches has its strengths. Therefore, all the approaches mentioned above were integrated in the development of OPTIS at different times.

Firstly, OPTIS was based on literature-derived teaching principles. In doing so, following the recommendations of Praetorius et al. [26,41], an inventory of commonly mentioned teaching principles from the literature was compiled. These evidence-based principles were added with quality criteria (“teaching principles”) needed to reflect the constructivist understanding of learning (theoretical foundation) that OPTIS follows (Table in S1 Table). This approach resulted in a first list of six dimensions of teaching and 27 teaching principles as a first version of OPTIS. After compiling this initial list of pertinent teaching principles, it was necessary to describe them with observable categories (**observation categories**). Providing well-described observation categories for each teaching principle was deemed necessary, as the close interrelation among different teaching principles could otherwise make it difficult to distinguish between them [40]. Therefore, existing, available, and validated observation protocols were reviewed, and three protocols were selected for specifying the observation categories: ELL [66], UBB [48], and an unpublished protocol from the MOVEOUT project [79]. The previously described protocol TEACH [4] was not used, as its intended purpose and origin differed too much from the aims of the present study. ELL [66] aligns with the intended purpose and therefore served as an inspiration for the observation categories in OPTIS. Furthermore, observation categories from the observation protocol UBB [48] helped to describe the literature-based teaching principles with observable categories. With the help of these two protocols, 16 of the 27 teaching principles could be described with observation categories. For the remaining eleven teaching principles, the research team formulated observable descriptions, based on the theoretical understanding of each teaching principle. After establishing the content of OPTIS, insights from an unpublished observation protocol from the project MOVEOUT [79] inspired the make-up of OPTIS. Aspects, such as summarizing all information on a single page and including additional details – such as “time,” “subject,” and “teacher” – were incorporated based on this protocol.

Subsequently, a **user manual** with examples for each observation category was developed. Content, design, and approach from Helmke [80] inspired the user manual of OPTIS. Each observation category was described with examples, which should help the observer to rate the extent to which the respective teaching principles can be observed (see the user manual in the appendix S2, which, in its current state, is only available in German).

In addition, the developmental progression included a determination of **observation (time) intervals**, which will structure observations using OPTIS. For observation intervals, different observation periods are commonly used during observational practice. The influence and importance of the length of observation intervals are discussed by Mashburn et al. [81]. Different observation intervals can vary from short snapshots of a few seconds [82–85] to intervals of one hour [75]. Helmke et al. [86] propose to use episodes, structured by the change of a specific didactic function or the applied social form. To target the goals of OPTIS to analyze teaching and gather as much information as possible, it appears appropriate to utilize applied social forms to define observation intervals, as different social forms promote the use of different teaching principles. Therefore, observations with OPTIS are structured with the help of five social forms (individual work, partner work, group work, plenum, and frontal teaching). Thus, each time the social forms changes, a new observation interval begins.

Regarding the application of **rating scales** in observing instructional quality, common practice varies from binary scales (e.g., [75]) to seven-point scales (e.g., [4,87]), with four-point (e.g., [88]) or five-point scales (e.g., [66]) being the most frequently used. Increasing the number of rating options allows for finer differentiation between observed behaviors; however, in real-time observation, more options make it more challenging to assign ratings quickly. Additionally, odd-numbered scales tend to encourage raters to select the middle option, while the even-numbered scale encourages raters to make more decisive judgments. Helmke adds another option, by including a fifth rating option, “not observable,” to his four-point ELL observation protocol. During the test phase, OPTIS was applied using a five-point rating scale, following Helmke’s recommendation to include the “not observable” option. In the final piloting of OPTIS, a six-point scale, also frequently used in the literature (e.g., [48]), was additionally applied to compare different approaches. The results of this comparison are reported in the Results section.

#### Step 2 – Testing and adjusting OPTIS based on expert feedback and real-time teaching observation.

At several points during the development process, experts were engaged in reviewing the observation protocol and provided ongoing verbal and written feedback to clarify the initial list of teaching principles and their observation categories. The first version of OPTIS was sent to a multi-disciplinary group of experts, consisting of teachers (n = 3), experts from education development practice (n = 2), educational research (n = 3) and observation specialists (n = 1). This diverse composition ensured that both practical and theoretical perspectives were considered, allowing the instrument to reflect the needs of practical teaching aspects while maintaining a strong scientific foundation. In July 2023, OPTIS went into its first test phase. During these field tests, OPTIS was tested by two independent observers in four different elementary schools in Bavaria, Germany. Refining the protocol’s categories and testing the usability during real-time observation were the main objectives of these first school observations. Furthermore, the user manual was supplemented with additional examples for each observation category drawn from those observations. The extension of the user manual was necessary to create the observer training later on. After completing the test phase, learnings from using OPTIS also led to rephrasing and modifying some observation categories.

Based on the experiences made during the test phase, the minimum length of observation intervals was restricted to five minutes, as Helmke [89] suggested. If social forms are changed within less than five minutes, the observation protocol remains unchanged, and the observation interval stays the same. However, the alteration in social form should be documented in the comment section, which is placed below the rating scheme. Additionally, in this comment section, the content of the teaching and the methods used by the teacher should be written down. This section was added due to recommendations from Praetorius et al. [41] that indicated that teaching principles varied in dependence on objectives, contents, and methods of teaching.

Within the second step, the **observer training** was developed. In previous observational practice, the duration of observer trainings varies widely, ranging from just a few hours to two weeks [21,70]. Throughout the development process, various training methods were evaluated (e.g., individual engagement with OPTIS and discussions during observation training with video material), leading to the design of a comprehensive three-step observer training program spanning approximately eight hours. This observer training starts with an individual confrontation with the observation protocol and associated user manual. A joint discussion followed once observers were confident in understanding each observation category and their differences. These first steps of creating a common theoretical understanding are essential for observer training [21,90]. During the test phase of the development of OPTIS, the observer training took five hours, only consisting of step one and three of the final observation training (it did not involve observations of video-recorded teaching). Reliability was computed after these training steps during the test phase. Reliability values were unsatisfying. Therefore, after establishing a common understanding of the observation items (first step of the training), watching video-recorded teaching was added to the observer training. The website https://www.fdz-bildung.de/videoportale provides a huge amount of freely accessible video-recorded classroom teaching for different subjects, teachers and grades. At least one video (45 minutes) should be observed during the observation training for using OPTIS (step two). Afterwards, observation ratings should be compared with other observers and discussed (step three). If necessary, more video-recorded teaching should be rated by using OPTIS, until the understanding of each teaching principle with its observation category is clear to all observers.

In October 2023, the test phase was followed by discussing and presenting OPTIS during a workshop in Switzerland, where experts from early childhood education and outdoor education met for discussing current practice and visions for the future. Especially the last teaching principles of OPTIS (learning environment, use of learning environment) as well as the criteria of instructional quality in tradition of the constructivism (e.g., action orientation, experience orientation) were discussed with experts (n = 9) from outdoor teaching and educational research. Besides this, many observation categories were revised and supplemented. After implementing the experts’ feedback and the learnings from the test phase, OPTIS consisted of 27 teaching principles with 39 observation categories. The amount of the teaching principles OPTIS consists of at this stage is equal to the test-phase, but two teaching principles were renamed. These principles and observation categories are displayed in Table 1. At this developmental stage, OPTIS went into the final piloting.

#### Step 3 – Piloting OPTIS with two independent and trained observers.

For the final piloting, two independent and trained observers rated video-recorded and real-time teaching from November 2023 to January 2024. During piloting, the two different rating scales were tested. Additionally, feasibility was continuously discussed and reliability of these two scales was calculated. First, ratings were realized using a five-point rating scale (like used by [66]). The five-point rating scale provides the rating options “does not apply”, “rather does not apply”, “rather does apply”, “does apply” and the option “not observable”. Further on in the piloting, observations with a six-point rating scale were piloted. The six-point rating scale includes the options +++ (“does apply completely”), ++ (“does apply”), + (“rather does apply”), – (“rather does not apply”), - - (“does not apply”), - - - (“does not apply at all”).

In this final stage, minor adaptations to OPTIS were made, mainly concerning the make-up of OPTIS. For example, the observation date was added to the heading of OPTIS or the articulation of observation categories were adjusted. The final version of OPTIS can be found in the appendix (S2 Table).

### Validation of OPTIS

#### Procedure.

During the test phase, real-time observations in four different elementary schools in Bavaria, Germany, were carried out. Two observers visited each school for one day and observed in total 13 school lessons by using the five-point rating scale. Because OPTIS was meant to be applicable subject-independently, data collections were realized across different subjects. For the test phase, lessons in mathematics, German, science, art, physical education and English were observed. The first author acted as one of the observers, the other observer was a university student.

The next phase, the piloting, started with observing video-recorded lessons. To be able to evaluate the intra-observer reliability, two observers rated 13 video-recorded school lessons twice. In this phase, the first author acted as one of the observers and a university student, different from the one during the test phase, acted as the second observer. The observed lessons were from different subjects (mathematics, German, physical education, French and science), took place in different grades (second to ninth grade) and were conducted by nine different teachers, across different cultures (Germany, Switzerland, and the UK). The average time between the first and the second rating amounted to two days. In total, both observers rated nine lessons by using the five-point rating scale and four lessons using the six-point rating scale. Two lessons were observed by both raters, using the five-point rating scale.

During this piloting, ten video-recorded school lessons and 13 real-time lessons were observed by the two observers from the intra-observer validation. The video-recorded lessons spanned from second to ninth grade, encompassing one lesson from second grade, one from third grade, two lessons from fourth grade, and one from fifth, sixth, seventh and ninth grade, respectively. The video-based piloting was implemented in the subjects of mathematics, German, science, physical education and French. During this piloting, the two observers rated six lessons with the five-point rating scale and four lessons using the six-point rating scale. For the real-time piloting, teaching was observed in a fourth grade in an elementary school in Bavaria, Germany. Lessons in mathematics, German and ethics were observed during these school visits. For the real-time piloting, the two observers rated three lessons using the five-point rating scale and ten lessons using the six-point rating scale.

#### Content validity.

To ascertain whether OPTIS really assesses instructional quality within the framework of a constructivist approach of learning, and to enhance and deliberate upon the content validity of OPTIS, 18 experts reviewed the observation protocol at different time points during the developmental process. The experts’ feedback was received in five consecutive rounds. The first round consisted of written feedback from three experts in the education section (one schoolteacher, one university teacher and one school seminar leader). They provided feedback on the general applicability of OPTIS and were asked to eliminate or add further teaching principles to the initial list. The second round, involving three experts from school and university teaching, was dedicated to feedback concerning the connection of teaching principles with their observation categories. Further, the experts were asked to identify duplicates among the observation categories. This expert round started with an independent analysis of each expert of the observation protocol and ended with an open online discussion guided by the main author. In the third round, the resulting version of OPTIS was reviewed by one teacher, with specific regard to the applicability in school. This third round was then followed by the test phase of the first version of OPTIS. This test phase was followed by the fourth expert round with two experts from educational and school observational research. They provided written and oral feedback about the extent to which the observation categories can measure the respective teaching principle they describe. Additionally, the rating scales were discussed. The resulting version of OPTIS was then presented during a workshop with nine experts from educational research, which represented the fifth round of evaluating the content validity. During the workshop, especially the constructivist understanding of teaching and the associated teaching principles were in the focus of discussion. Finally, the observability of each observation category was checked during the final video and real-time piloting.

#### Data analysis.

In accordance with classical test theory, an observation protocol should be examined with regard to the traditional criteria of objectivity, reliability, and validity to establish the foundation for defining the quality of an observation [48,91]. In data assessments using observations, the objectivity of the ratings is highly connected with the reliability of the observation*.* The intra-observer reliability refers to the consistency of observations made by a single observer across multiple time points. Inter-observer reliability refers to the agreement between two or more raters and measures the extent to which different raters provide similar judgements or scores for the same observations [91]. When analyzing classroom practice by means of observation, observer bias is considered as a crucial aspect [21,92]. To discuss this bias, inter- and intra-observer reliability was calculated by using Gwet’s Agreement Coefficient 2 [93,94]. In comparison to other commonly used reliability coefficients (e.g. Cohen’s Kappa or Krippendorff’s Alpha), Gwet’s AC2 is less affected by prevalence or marginal probability problems [95,96]. Contrarily, evaluating the inter- and intra-observer reliability with Gwet’s AC2 shows very stable reliability coefficients for observer agreement and may therefore serve to avoid these problems [93]. AC2 is based on the same underlying principles as AC1, but it differs in the calculation of the expected agreement. The formula for AC2 involves computing observed agreement and expected agreement adjusted for chance, similar to the calculation of AC1. However, AC1 only supports categorical data, whereas AC2 can be used when dealing with ordinal data [93].

Formula for calculation of Gwet’s AC2:


$$\begin{array}{c} AC\text{2}=\frac{P_{o}-P_{e}}{\text{1}-P_{e}} \end{array}$$

*P*_*o =*_ Observed agreement

*P*_*e =*_ Expected agreement adjusted for chance

The values of *P*_*o*_ and *P*_*e*_ are calculated based on the observed and expected frequencies of agreement, considering the overall distribution of ratings across all categories. This adjustment accounts for chance agreement and provides a reliable estimate of inter- and intra-observer reliability. Observation ratings were saved and preprocessed in Excel. Later, they were analyzed with Python. AC2 was computed using Fergadis [97] Python port of Gwet’s [98] irrCAC package for calculating chance-corrected agreement coefficients. Gwet’s AC2 scores can be interpreted using commonly utilized benchmarks, as for example Altman [99] proposed. Gwet’s AC2 has a minimum value of −1 (systematic disagreement among raters) and a maximum value of 1 (perfect agreement among raters). Perfect agreement means that there is complete consistency among raters with no variability due to chance. A value of 0 indicates no agreement beyond what would be expected by chance. In other words, the observed agreement is no better than what would be achieved randomly. Every value between 0 and 1 indicates agreement beyond chance, but less than perfect agreement. The closer the value is to 1, the higher the agreement among raters. Altman [99] suggests the following interpretation: values < 0.2 represent a slight agreement. If the value is between 0.20 ≤ AC2 < 0.4, there is fair agreement. For values of 0.40 ≤ AC2 < 0.6, there is moderate agreement; for 0.60 ≤ AC2 < 0.8, there is good agreement and AC2 values ≥ 0.80 indicate very good agreement. However, to interpret the AC2 coefficient the standard error was considered, as proposed by Gwet [100]. All data and results can be viewed in a Streamlit Data App (https://optis-irr.streamlit.app/).

## Results

Before presenting the results, it is important to note that due to the defined observation intervals depending on social form, different amounts of observation protocols were completed during different school lessons (45 minutes). Therefore, the amount of completed observation protocols varies between one and four per school lesson. On average, three observation protocols were employed during 45 minutes of teaching. Observation intervals varied from five minutes (which was defined as the minimum observation length) to 45 minutes.

### Reliability

Reliability coefficients were calculated across all observation categories (Table 2) as well as for the specific observation categories (Table 3).

**Table 2:** AC2 coefficients for intra- and inter-observer reliability during test phase and piloting.

			Rating Scale	AC2 coefficient	observation protocols (n)
**Intra-observer reliability**	Piloting	Video-based	5-point	*0.896 (rater 1)* *0.909 (rater 2)*	*21* *6*
			6-point	*0.898 (rater 1)* *0.974 (rater 2)*	*9* *3*
**Inter-observer reliability**	Test phase	Real-time	5-point	*0.595*	*32*
	Piloting	Video-based	5-point	*0.937*	*18*
			6-point	*0.929*	*12*
		Real-time	5-point	*0.903*	*7*
			6-point	*0.932*	*22*

**Table 3:** AC2 coefficients for inter-observer reliability during test phase and piloting for each teaching principle with its observation categories.

Teaching principles	Observation categories	Test phase	Piloting			
		School	Video		school	
		5 scale	5 scale	6 scale	5 scale	6 scale
**Action orientation**	Content is developed by the students through their own actions (these actions serve as a starting point for further teaching activities)	0,749	0,94	0,931	0,927	0,927
**Experience-based**	Students can gain their own experience in the classroom	0,613	0,981	0,971	0,989	0,955
**Holistic Approach**	Different senses of the students are addressed in class (learning with head, heart & hand)	0,635	0,985	0,945	0,986	0,94
**Competence orientation**	The significance of the subject matter is made clear to the students by the learning objectives (present significance/future significance)	0,766	0,982	0,948	1	0,924
	Students assess their performance and competencies themselves	/	0,858	0,858	0,901	0,936
**Motivation**	The students learning & performance needs are taken into account, awakened & maintained	0,668	0,942	0,935	0,969	0,964
**Target orientation**	Learning objectives are clearly formulated by the instructor & made clear to the students	0,819	0,928	0,927	0,878	0,962
**Process orientation**	The learning paths/the learning process is perceived as an important sub-component by the teacher	0,656	0,961	0,985	0,889	0,947
**Independence**	Independent learning is encouraged and challenged in a variety of ways in the classroom	0,445	0,963	0,923	0,961	0,936
**Learner orientation**	The teacher aligns the lessons to the interests and needs of the students	0,67	0,932	0,826	0,894	0,91
**Participation**	Students are given space to help shape and design the lesson	/	0,952	0,818	0,982	0,92
**Experiencing self-efficacy**	The lessons open up for individual learning and solutions	0,618	0,917	0,916	0,901	0,948
**Cooperative Learning**	Students support each other in their learning/ social interactions take place	0,105	0,9	0,791	0,98	0,906
**Variety of methods**	The teacher uses various methods to organize the lessons	0,809	0,988	0,96	0,961	0,965
**Visualization**	The learning processes are supported by the use of various media by the teacher	0,664	0,911	0,97	0,986	0,943
**Transparent performance expectations**	The instructor makes the requirements transparent to the students/The students know what is expected of them at all times	0,677	0,98	0,987	0,97	0,949
**Intelligent practice**	The exercise phases fit in with the lessons	/	0,98	0,994	0,976	0,99
**Fuse of content**	What has been learned is practiced, repeated, deepened and secured sufficiently often by the students	0,744	0,895	0,913	0,81	0,966
**Differentiation**	The LK supports different learning speeds, learning paths & performance levels	0,456	0,926	0,935	0,986	0,838
	Students receive differentiated feedback and assistance	0,41	0,868	0,912	0,851	0,868
**Activate prior knowledge**	The content can be linked to previous knowledge and experience	0,801	0,994	0,976	0,791	0,946
**Interdisciplinary**	There is a transfer between different subjects	0,538	0,958	0,964	0,966	0,997
**Authentic & problem-based learning experiences**	The lessons contain examples from everyday life/ from the students’ living environment/ address problems	0,663	0,968	0,896	0,947	0,801
**Structure & clarity**	The lessons have a common thread (lesson content builds on each other)	0,637	0,996	0,994	0,867	0,969
	The teaching process is based on recognizable rules	0,407	0,996	1	1	0,986
**Time utilization** (High proportion of real learning time)	Answers of the students or work phases are not interrupted by the teacher	0,862	0,796	0,94	0,511	0,965
	Students are busy with learning activities/lesson-related content almost all the time	0,785	0,972	0,862	0,976	0,98
**Communication**	The volume is appropriate to the social form & learning content	0,974	0,996	0,994	0,989	0,997
	The teacher works with non-verbal communication (gestures/mimic/rituals)	0,844	0,983	0,952	0,989	0,997
	Work assignments, explanations & impulses are formulated clearly, precisely & coherently	/	0,986	1	0,966	0,993
**Learning-friendly** **classroom climate**	The teacher recognizes and encourages the students’ willingness to learn	0,831	0,95	0,994	0,966	0,968
	Dealing with mistakes in a way that promotes learning/ students’ contributions are seen as learning opportunities	0,825	0,885	0,922	0,98	0,939
	The teacher encourages the students to ask questions/express their thoughts	0,511	0,772	0,904	0,83	0,936
	There is an atmosphere of mutual appreciation and respect between teachers and students	0,829	1	0,994	1	0,997
	…, and between students and students	/	0,996	0,993	0,959	0,997
**Learning environment**	The choice of learning environment matches with the teaching topic	0,889	0,954	0,93	0,927	0,934
	The learning environment matches the learning phase	0,679	0,973	0,926	0,966	0,934
	Students explore the learning environment through movement and interaction	0,854	0,985	0,979	0,968	0,986
**Use of the learning environment**	The learning environment is actively integrated into the lesson (it becomes the departure of learning)	0,688	0,99	0,982	1	1
**AC2 across all observation categories**		**0,595**	**0,937**	**0,929**	**0,903**	**0,932**

Note. The values represent AC2 coefficients for each observation category.

“/” means that these observation categories did not exist at that stage of development.

#### Intra-observer reliability.

When using the five-point rating scale, the overall coefficient for Gwet’s AC2 across both observers was 0.898. With regard to the different observation categories, the AC2 coefficient varied between 0.514 and 1. For observations using the six-point rating scale, the overall AC2 coefficient across both observers was 0.912. For the different observation categories, the AC2 coefficient varied between 0.744 and 1. For the two lessons both observers rated twice, the AC2 coefficient for the first observer was 0.879 and for the second observer 0.909, using the five-point rating scale.

#### Inter-observer reliability.

*Test phase*: The inter-observer reliability was computed at several times during the developmental process of OPTIS. During the test phase, the AC2 coefficient across the 36 observation categories varied between 0.105 and 0.974, with an average of 0.595. During this test phase, the teaching principle *cooperative learning* achieved poor agreement (0.105) beyond what would be expected by chance alone. The teaching principles *differentiation* (0.456/0.410) and *independence* (0.445) as well as the observation category *the teaching process is based on recognizable rules* (0.407) achieved moderate agreement between the raters. All the other observation categories achieved AC2 coefficients above 0.5 during the test phase, which indicated moderate to good agreement.

*Piloting*: During the video-based piloting, the observation protocol consisted of 39 observation categories, which represented the final version of OPTIS (Table in S2 Protocol). For the five-point rating scale, the AC2 ranged between 0.772 and 1, with an overall AC2 coefficient across all categories of 0.937. For the six-point rating scale, the AC2 values were between 0.791 and 1 with an overall AC2 coefficient of 0.929. For the piloting based on real-time observations, the overall coefficient for the five-point rating scale was 0.903 (0.511–1) and for the six-point rating scale 0.932 (0.801–1) (see Table 2). The values for the different teaching principles with their observation categories are documented in Table 3.

*Content validity*: To guarantee content validity, OPTIS was developed based on commonly mentioned teaching principles, added with teaching principles to reflect the constructivist learning theory (see Table in S1 Table/Fig 1). Furthermore, OPTIS was continuously discussed with experts during the whole development process. The chosen teaching principles and their observation categories underwent repeated evaluation, drawing upon theoretical foundations, empirical evidence, and the expertise of the involved experts. Due to these discussions, the content validity of OPTIS could be improved continuously. Final adjustments were made due to the observations during the field tests and video-recorded teaching. Finally, the content validity of OPTIS is considered given based on the experts’ feedback, as well as the evaluation and the adjustments made throughout the process (e.g., [77,82] 7).

## Discussion

Monitoring instructional quality is widely regarded as the most influential factor to explain a considerable portion of variance in student development and achievements [3–7]. To monitor instructional quality, classroom measurements such as structured classroom observation should be supported by reliable and valid observation instruments. Current available observation protocols are facing several limitations concerning aims, theoretical foundation and biases [21,92]. By developing OPTIS, the aim was not to define the characteristics of “good” teaching. Instead, the objective was to develop an observation protocol to identify the applied teaching principles during lessons, so that teachers can monitor teaching and learning processes by external observers and, in a subsequent step, can reflect on their own teaching processes.

To evaluate the quality of the developed observation protocol, intra- and inter-observer reliability were computed at different stages during the developmental process. The inter-observer reliability resulting from the test phase suggested a considerable level of consistency and reliability of ratings, indicating that the raters generally agree more than they would by chance (Table 2). However, the test phase also revealed weaknesses concerning the reliability of OPTIS at this stage, which were reflected in low AC2 values for some observation categories (Table 3). To further reduce rater biases, as low or moderate AC2 values indicate, observer trainings can help to minimize the subjective biases of raters [5,8,64,101]. Therefore, during the developmental process, an observer training was developed, which was not used during the test phase (see step two). As Hoyt and Kerns [102] indicated, the length of observer training does not affect the quality of the observer ratings. By implementing a three-step, eight-hour observer training, we opt for a duration situated midway between common established observer trainings (5–24 hours). During the final piloting, the observer training was applied to train both observers. By applying this observer training, good to very good reliability values could be achieved. Therefore, to further use OPTIS, this training, as recommended by the research team, should be realized before using OPTIS.

As demonstrated by Drexl [48] or Carifio and Perla [103], reliability checks are necessary to validate observation protocols. Ladics et al. [70] showed in their review that only a few observation protocols provide measurements for reliability. Eleven of the 20 included studies provided some data concerning reliability. However, only three of them demonstrated that they computed reliability. If reliability is checked, very different indices are commonly used for computing reliability: Cohen’s Kappa, intraclass correlation coefficient (ICC) or Krippendorff’s Alpha [104–107]. As presented by Wals et al. [108] or Hallgren [109], different inter-observer measures serve different purposes. The initial identification of the appropriate measure for the data under investigation is perhaps the most crucial step when computing inter-observer reliability [108]. Employing Cohen’s Kappa or Krippendorff’s Alpha may result in the paradox where a genuinely high level of observer agreement could yield low reliability values [110,111]. As demonstrated by, for example, Wongpakaran et al. [96], Gwet’s AC2 is less affected by prevalence or marginal probability problems and may therefore serve to avoid these problems [93,95]. In educational research, Gwet’s AC2 is rarely computed, even though evidence indicates that for ordinal data, small samples and uneven distribution of ratings, AC2 seems to be an applicable and stable measure [96]. Due to our experience dealing with such data, reliability calculation by using the AC2 agreement can be highly recommended. In educational research, reliability checks are often (if) only being realized for a small proportion of the complete sample (as, e.g., Molina et al. [4], where reliability is only checked for 20% of the data). Therefore, reliability checks in educational research often deal with small samples. In this case, AC2 is regarded as a stable measurement [93].

For the real-time piloting, the observation protocols applied with the six-point rating scale could achieve slightly better inter-observer reliability values (0.801 to 1) than the observations with the five-point rating scale (0.511 to 1). Various discussions about the best choice of rating scales appeared over time and findings are still contradictory [103,112,113]. A six-point rating scale provides more response options. It allows respondents to express a wider range of opinions or attitudes and can thus capture subtler variations in ratings and help prevent response biases. This increased granularity can result in more precise measurements and potentially reduce the clustering effect often seen in rating scales with fewer response options [112,114]. During the piloting, both observers appreciated the more precise rating options with the six-point rating scale. Additionally, both observers had problems in differentiating between the rating option ‘not applied’ and ‘not observable’, while using the five-point rating scale.

Concerning the observation time intervals, the change of social forms was used as a prompt for a new observation interval and protocol. Commonly applied time intervals differ largely [75,82–84]. Cronbach [115] suggests to define the scope to reduce measurement errors, which means to clearly subdivide the observation intervals. Meyer et al. [116] identified the dynamic and complexity of teaching as the most problematic source of measurement errors. Therefore, minimizing these complex situations of teaching can help to improve subsequent results. The applied teaching principles vary greatly depending on the different social forms, and therefore, social forms, as visual dimensions of teaching, can significantly enhance the structure of instruction [34]. The minimum of five minutes’ observation interval was confirmed during the piloting. OPTIS consists of 39 observation categories. To observe and rate all of them, five minutes are considered the least amount of time. Due to the learnings from the piloting and some results from inter- and intra-observer reliability calculations, we recommend to use OPTIS by applying the six-point rating scale and using the change of social forms to define observation time intervals.

The real-time piloting was mainly realized using the six-point rating scale (n = 22). For this piloting, good to very good agreement beyond what would be expected by chance alone among raters was achieved (0.801 to 1). This suggests a high level of consistency and reliability within the observation ratings. The lowest value during this real-time piloting with the six-point rating scale was computed for the teaching principle ‘*authentic & problem-based learning experiences’* with its observation category ‘*the lessons contain examples from everyday life/ from the students’ living environment/ address problems’*. This value might result from the description of its observation category. The observation category comprises two aspects. One aspect is the connection to the life of the learner (authentic), the second aspect is the problem-based aspect of this category. During the observation, the observer has to decide on which aspect of this category he/she is focusing on. This might result in lower agreement values. Furthermore, problem-based learning goes back to Dewey [117] and is closely connected to other teaching principles within OPTIS, such as ‘*learner orientation’* or ‘*experience orientation’*. This demonstrates various concepts behind problem-based learning in research. Another problem arose during real-time observation. There, the two observers had disagreements about authentic learning examples. Do examples from a teaching book that focuses on children, perhaps of the same age as the learners, truly represent authentic examples? Why talk about other children of the same age, which represent semi-authentic examples, instead of taking examples from the learner’s group, i.e., real authentic examples? Resulting from these problems, this observation category should be further detailed in the subsequent use of OPTIS. One opportunity could be reducing the category to ‘*the lessons contain examples from everyday life’.* The aspect ‘*address problems’* can be regarded as covered by the teaching principle ‘*learner orientation’*. All the other observation categories could achieve AC2 values above 0.83, which indicates very good agreement among raters and suggests a clear and consistent understanding among different observers.

Concerning the validity of OPTIS, extensive discussions and several rounds of expert feedback, as well as the test- and piloting phase helped to improve the content validity over the whole process. Although the recruiting of the involved experts did not follow a strict procedure (unlike Leff et al. [59], who did by using Participatory Action Research or Elmendorf and Song [77] using a Delphi-Method), various experts could be included during the developmental process of OPTIS. Additionally, different methods of expert feedback (verbal and written) helped to sustainably improve the validity of OPTIS. During verbal feedback, questions or unclear wordings could be discussed in detail, whereas feedback provided in written form gave the experts the chance to digest information at their own pace and reflect on it. Additionally, written feedback was captured, and the author team could easily refer to it at several times during the process. The mixture of provided feedback and the two application phases strengthened the validity of OPTIS. Based on these results, the developmental process with all its steps and adaptations can be regarded as successful and can be recommended for the development of future observation protocols.

### Limitations

The strength of several expert rounds and field tests to develop and improve OPTIS is followed by a limitation regarding the validity of OPTIS and its purpose. As detailed in the introduction, due to the vast amount of available teaching principles, it is nearly impossible to provide a comprehensive comparison of all available teaching principles (e.g., [5,36,118]). By developing OPTIS, the authors tried to work collaboratively (as recommended by Charalambous et al. [119]) to create a large agreement about the included teaching principles and their understanding. However, the background of available teaching principles on which OPTIS was based can still only be regarded as limited and, to some extent, subjective by the research team.

To allow a subject-independent usage of OPTIS, lessons in different subjects (mathematics, German, science, physical education, ethics and French) were observed. Nevertheless, each subject was only rated a few times. The same situation occurs regarding the independent application of OPTIS by different teachers and in different grades. For the video-based observations, classes between second and ninth grade were observed. Each grade, and therefore each teacher, was observed only once during these video observations. For the real-time observations during the piloting, only one class, being taught by one teacher, was observed. In summary, the sample size was small. We assume that to support subject-, grade-, and teacher independency of OPTIS, further studies are needed. Besides these limitations, OPTIS has not yet been evaluated in a cross-cultural context. However, it must be noted that experts from Germany and Switzerland provided feedback, and in the video-based piloting, OPTIS was tested with videos from different countries, like the UK or Switzerland. Yet, in the real-time observation, only German schooling could be observed. Observation intervals for OPTIS are based on the change of the social form. Therefore, regardless of how the teaching is structured (time intervals) or the type of instruction used (more frontal or group teaching), OPTIS appears to be applicable. But, as we could not test OPTIS by real-time observations in different cultural samples, these assumptions are to be verified in the future.

Additionally, to receive a multi-perspective state of the observed teaching, other perspectives, like the ones of students and teachers themselves, should be included [38]. To deeply analyze teaching, it is crucial to consider the perspective of students. This provides valuable insight to further analyze learning processes and applied teaching principles.

## Supporting information

S1 Table Literature-based selection of the teaching principles the observation protocol is based on. (DOCX)

S2 Table Observation protocol – OPTIS. In German language. (DOCX)
