# Supplementary material for: Observing teaching principles in school - Development and Validation of an Observation Protocol (OPTIS)
Source: PLoS One. 2026 Aug 17;21(8):e0355710. doi: 10.1371/journal.pone.0355710 (PMC13480659; doi:10.1371/journal.pone.0355710)
Supplement: Supplementary file 1 [file pone.0355710.s001.docx]

**S1 Table. Literature-based selection of the teaching principles the observation protocol is based on.**

|  | **Teaching principles** | **Literature** |
| --- | --- | --- |
| **(Cognitive)**  **activation**  (1) | **Action orientation** | Wiater (2); Pieper and Krawinkel (3); Tulodziecki, Herzig (4); Gerstenmaier and Mandl (5); Berliner (6) |
|  | **Experience-based** | Pieper and Krawinkel (3); Gerstenmaier and Mandl (5) |
|  | **Holistic approach** | Wiater (7), Pieper and Krawinkel (3); Rickinson (8) |
|  | **Competence orientation** | Wiater (2); Charalambous and Praetorius (9); Kultus Minister Konferenz (KMK) (10); Brophy and Alleman (11) |
|  | **Motivation** | Wiater (2); Charalambous and Praetorius (9); Kiel (12); Helmke (13); Kultus Minister Konferenz (KMK) (10); Ditton (14) |
|  | **Target orientation** | Wiater (2); Wiater (7); Charalambous and Praetorius (9); Kiel (12); Kunter (15); Helmke (13); Schilmöller (16); Brophy (17) |
| **Lesson**  **design** | **Process orientation** | Kultus Minister Konferenz (KMK) (10); Brophy (17) |
|  | **Independence** | Wiater (2); Helmke (13); Kultus Minister Konferenz (KMK) (10) |
|  | **Learner orientation** | Wiater (2); Kiel (12); Helmke (13); Kultus Minister Konferenz (KMK) (10) |
|  | **Participation** | Charalambous and Praetorius (9); Meyer (18) |
|  | **Experiencing self-efficacy** | Kultus Minister Konferenz (KMK) (10); Deci (19) |
|  | **Cooperative learning** | Charalambous and Praetorius (9); Muijs and Reynolds (20); Brophy (17); Slavin (21) |
|  | **Variety of methods** | Charalambous and Praetorius (9); Helmke (13); Kunter (15); Meyer (22) |
|  | **Visualization** | Wiater (2); Charalambous and Praetorius (9); Kiel (12); Ditton (14) |
|  | **Transparent performance expectations** | Schilmöller (16), 2010; Meyer (22), Brophy (17) |
|  | **Intelligent practice** | Charalambous and Praetorius (9); Helmke (13); Meyer (22) |
|  | **Fuse of content** | Wiater (2); Charalambous and Praetorius (9); Helmke (13) |
|  | **Differentiation** | Wiater (2); Charalambous and Praetorius (9); Kiel (12); Helmke (13); Kunter (15); Hattie and Timperley (23); Meyer (22) |
| **Contents & Topics** | **Activation of prior knowledge** | Kunter (15); Kultus Minister Konferenz (KMK) (10); Brophy (17); Ditton (14), Gerstenmaier and Mandl (5) |
|  | **Interdisciplinary** | Roßbach (24); Gerstenmaier and Mandl (5) |
|  | **Authentic and problem-based learning**  **experiences** | Charalambous and Praetorius (9); Kunter (15); Kultus Minister Konferenz (KMK) (10); Schilmöller (16); Brody (25), Roßbach (24); Bredo (26); Zimmerman (27) |
| **Classroom**  **management**  (1) | **Structure and clarity** | Wiater (2); Charalambous and Praetorius (9); Kiel (12); Helmke (13); Meyer (22); Brophy (17); Ditton (14); Hattie (28) |
|  | **Time utilization (high proportion of real learning time)** | Charalambous and Praetorius (9); Kiel (12); Helmke (13); Kunter (15); Muijs and Reynolds (20); Meyer (22); Ditton (14) |
|  | **Communication** | Charalambous and Praetorius (9); Meyer (22); Brophy (17); Ditton (14) |
| **Constructive**  **support**  (1) | **Learning-friendly**  **classroom climate** | Charalambous and Praetorius (9); Helmke (13); Kunter (15); Schilmöller (16); Clausen (29); Brophy and Alleman (11) |
| **Learning space** | **Learning environment** | Badri, Yang (30); Meyer (22); Roßbach (24); Gerstenmaier and Mandl (5) |
|  | **Use of the learning**  **environment** |  |

Note. This table presents the literature on which the initial collection of teaching principles for the first version of OPTIS was based. In addition, it includes literature supporting the quality criteria (“teaching principles”) added to represent constructivist learning theory.

**References**

1. Klieme E, Schümer G, Knoll S. Mathematikunterricht in der Sekundarstufe I: Aufgabenkultur und Unterrichtsgestaltung. TIMSS - Impulse für Schule und Unterricht. 2001:43–57.

2. Wiater W. Unterrichtsprinzipien: Prüfungswissen - Basiswissen Schulpädagogik. 8th ed. Donauwörth: Auer; 2021.

3. Pieper U, Krawinkel U. Handlungsorientiertes Lernen an der Albert-Schweitzer-Schule Münster. In: Fischer C, Schilmöller R, editors. Was ist guter Unterricht? Qualitätskriterien auf dem Prüfstand. Münster: Aschendorff; 2010. p. 169–75.

4. Tulodziecki G, Herzig B, Blömeke S. Gestaltung von Unterricht. Eine Einführungin die Didaktik. Bad Heilbrunn: Klinkhardt.; 2009.

5. Gerstenmaier J, Mandl H. Wissenserwerb unter konstruktivistischer Perspektive. Zeitschrift für Pädagogik. 1995; 41:867–88.

6. Berliner DC. Telling the stories of educational psychology. Educational Psychologist. 1992;27:143–61.

7. Wiater W. Unterrichtsprinzipien. Augsburg: Auer; 2018. 136 p.

8. Rickinson M, Dillon J, Teamey K, Morris M, Choi M, Sanders K, and Benefield P. A review of research on outdoor learning. 2004.

9. Charalambous CY, Praetorius A-K. Creating a forum for researching teaching and its quality more synergistically. Studies in Educational Evaluation. 2020;67:100894.

10. Kultus Minister Konferenz (KMK). Konzeption der Kultusministerkonferenz zur Nutzung der Bildungsstandards für die Unterrichtsentwicklung. KMK: 2010.

11. Brophy J, Alleman J. Activities as instructional tools: A framework for analysis and evaluation. Educational researcher. 1991;20(4):9–23.

12. Kiel E. Unterricht sehen, analysieren und gestalten. Bad Heilbrunn: Verlag Julius Klinkhardt; 2018.

13. Helmke A. Unterrichtsqualität und Lehrerprofessionalität. Diagnose, Evaluation und Verbesserung des Unterrichts. 5 ed: Klett; 2014.

14. Ditton H. Qualitätskontrolle und Qualitätssicherung in der Schule und Unterricht. Ein Überblick zum Stand der empirischen Forschung. In: Helmke A, Hornstein W, Terhart E, editors. Qualität und Qualitätssicherung im Bildungsbereich; Schule, Sozialpädagogik, Hochschule. Zeitschrift für Pädagogik. 41. Weinheim: Beltz; 2000. p. 73–92.

15. Kunter MT, Ulrich. Psychologie des Unterrichts. Ferdinand Schöningh: Paderborn; 2013.

16. Schilmöller R. Guter Unterricht - Gute Schule. Notwendige Kontexte gelingender Unterrichtspraxis. In: Schilmöller R, Fischer C, editors. Was is guter Unterricht? Qualitätskriterien auf dem Prüfstand. Münster: Aschendorff; 2010.

17. Brophy J. Teaching. Educational Practices Series-1. 2000.

18. Meyer H. Was ist guter Unterricht? Berlin: Cornelsen; 2020. 192 Seiten p.

19. Deci EL, Ryan RM. The “What” and “Why” of Goal Pursuits: Human Needs and the Self-Determination of Behavior. Psychological Inquiry. 2000;11:227–68.

20. Muijs D, Reynolds D. effective teaching. evidence and practice. London: Sage; 2005.

21. Slavin RE. Cooperative Learning: Theory, Research, and Practice: Prentice Hall; 1990.

22. Meyer H. Zehn Merkmale guten Unterrichts. Empirische Befunde und didaktische Ratschläge. Pädagogik. 2003;10:36–43.

23. Hattie J, Timperley H. The Power of Feedback. Review of Educational Research. 2007;77(1):81–112.

24. Roßbach H-G. Unterrichtsqualität im 2. Schuljahr - Ergebnisse einer empirischen Untersuchung. Unterrichtswissenschaft. 2002;30:230–45.

25. Brody M. Learning in nature. Environmental Education Research. 2005;11.

26. Bredo E. Reconstructing educational psychology: situated Cognition and Deweyian pragmatism. Educational psychologist. 1994;29:23–35.

27. Zimmerman BJ. Self-regulated learning and academic achievement: An overview. Educational Psychologist. 1990;25:3–17.

28. Hattie J. Visible learning: A synthesis of meta-analyses relating to achievement. London [u.a.]: Routledge; 2009. IX, 378 p.

29. Clausen M. Unterrichtsqualität: Eine Frage der Perspektive? Münster: Waxmann; 2002.

30. Badri M, Yang G, Al Mazroui K, Mohaidat J, Al Rashedi A, Al Housani N. Out-of-school experience categories influencing interest in biology of secondary school students by gender: exploration on an Abu Dhabi sample. Journal of Biological Education. 2016;51(2):166–85.

1.	Klieme E, Schümer G, Knoll S. Mathematikunterricht in der Sekundarstufe I: Aufgabenkultur und Unterrichtsgestaltung. TIMSS - Impulse für Schule und Unterricht. 2001:43–57.

2.	Wiater W. Unterrichtsprinzipien: Prüfungswissen - Basiswissen Schulpädagogik. 8. Auflage ed. Donauwörth: Auer; 2021.

3.	Pieper U, Krawinkel U. Handlungsorientiertes Lernen an der Albert-Schweitzer-Schule Münster. In: Fischer C, Schilmöller R, editors. Was ist guter Unterricht? Qualitätskriterien auf dem Prüfstand. Münster: Aschendorff; 2010. p. 169–75.

4.	Tulodziecki G, Herzig B, Blömeke S. Gestaltung von Unterricht. Eine Einführung

in die Didaktik. Bad Heilbrunn: Klinkhardt.; 2009.

5.	Gerstenmaier J, Mandl H. Wissenserwerb unter konstruktivistischer Perspektive. Zeitschrift für Pädagogik. 1995;41:867–88.

6.	Berliner DC. Telling the stories of educational psychology. Educational Psychologist. 1992;27:143–61.

7.	Wiater W. Unterrichtsprinzipien. Augsburg: Auer; 2018. 136 Seiten p.

8.	Rickinson M, J. Dillon, K. Teamey, M. Morris, M. Choi, K. Sanders, and P. Benefield. A review of research on outdoor learning. 2004.

9.	Charalambous CY, Praetorius A-K. Creating a forum for researching teaching and its quality more synergistically. Studies in Educational Evaluation. 2020;67:100894.

10.	Kultus Minister Konferenz (KMK). Konzeption der Kultusministerkonferenz zur Nutzung der Bildungsstandards für die Unterrichtsentwicklung 2010.

11.	Brophy J, Alleman J. Activities as instructional tools: A framework for analysis and evaluation. Educational researcher. 1991;20(4):9–23.

12.	Kiel E. Unterricht sehen, analysieren und gestalten. Bad Heilbrunn: Verlag Julius Klinkhardt; 2018.

13.	Helmke A. Unterrichtsqualität und Lehrerprofessionalität. Diagnose, Evaluation und Verbesserung des Unterrichts. 5 ed: Klett; 2014.

14.	Ditton H. Qualitätskontrolle und Qualitätssicherung in der Schule und Unterricht. Ein Überblick zum Stand der empirischen Forschung. In: Helmke A, Hornstein W, Terhart E, editors. Qualität und Qualitätssicherung im Bildungsbereich; Schule, Sozialpädagogik, Hochschule. Zeitschrift für Pädagogik. 41. Weinheim: Beltz; 2000. p. 73–92.

15.	Kunter MT, Ulrich. Psychologie des Unterrichts. Ferdinand Schöningh: Paderborn; 2013.

16.	Schilmöller R. Guter Unterricht - Gute Schule. Notwendige Kontexte gelingender Unterrichtspraxis. In: Schilmöller R, Fischer C, editors. Was is guter Unterricht? Qualitätskriterien auf dem Prüfstand. Münster: Aschendorff; 2010.

17.	Brophy J. Teaching. Educational Practices Series--1. 2000.

18.	Meyer H. Was ist guter Unterricht? Berlin: Cornelsen; 2020. 192 Seiten p.

19.	Deci ELR, Richard M. The “What” and “Why” of Goal Pursuits: Human Needs and the Self-Determination of Behavior. Psychological Inquiry. 2000;11:227–68.

20.	Muijs D, Reynolds D. effective teaching. evidence and practice. London: Sage; 2005.

21.	Slavin RE. Cooperative Learning: Theory, Research, and Practice: Prentice Hall; 1990.

22.	Meyer H. Zehn Merkmale guten Unterrichts. Empirische Befunde und didaktische Ratschläge. Pädagogik. 2003;10:36–43.

23.	Hattie J, Timperley H. The Power of Feedback. Review of Educational Research. 2007;77(1):81–112.

24.	Roßbach H-G. Unterrichtsqualität im 2. Schuljahr - Ergebnisse einer empirischen Untersuchung. Unterrichtswissenschaft. 2002;30:230–45.

25.	Brody M. Learning in nature. Environmental Education Research. 2005;11.

26.	Bredo E. Reconstructing educational psychology: situated Cognition and Deweyian pragmatism. Educational psychologist. 1994;29:23–35.

27.	Zimmerman BJ. Self-regulated learning and academic achievement: An overview. Educational Psychologist. 1990;25:3–17.

28.	Hattie J. Visible learning: A synthesis of meta-analyses relating to achievement. London [u.a.]: Routledge; 2009. IX, 378 S. p.

29.	Clausen M. Unterrichtsqualität: Eine Frage der Perspektive? Münster: Waxmann; 2002.

30.	Badri M, Yang G, Al Mazroui K, Mohaidat J, Al Rashedi A, Al Housani N. Out-of-school experience categories influencing interest in biology of secondary school students by gender: exploration on an Abu Dhabi sample. Journal of Biological Education. 2016;51(2):166–85.
