# Supplementary material for: Observing teaching principles in school - Development and Validation of an Observation Protocol (OPTIS)
Source: PLoS One. 2026 Aug 17;21(8):e0355710. doi: 10.1371/journal.pone.0355710 (PMC13480659; doi:10.1371/journal.pone.0355710)
Supplement: Supplementary file 2 [file pone.0355710.s002.docx]

***Beobachtungsprotokoll (OPTIS)***	Datum: ____________	Nr.___________

| Klasse | Lehrkraft | Unterrichtsfach | Sozialform (F/P/GA/PA/EA)* | Zeit (Start) | Zeit (Ende) | Lernort |
| --- | --- | --- | --- | --- | --- | --- |
|  |  |  |  |  |  |  |

|  | Unterrichtsprinzipien | Beobachtungskategorien | **+ + +** | **+ +** | **+** | **-** | **- -** | **- - -** |
| --- | --- | --- | --- | --- | --- | --- | --- | --- |
| **(Kognitive) Aktivierung** | **Handlungsorientierung** | Inhalte werden von den SuS durch eigenes Handeln erschlossen (dieses Handeln dient als Ausgangspunkt für weiteres unterrichtliches Handeln) |  |  |  |  |  |  |
|  | **Erfahrungsorientierung** | Die SuS können eigene Erfahrungen im Unterricht sammeln |  |  |  |  |  |  |
|  | **Ganzheitlichkeit** | Verschiedene Sinne der SuS werden im Unterricht angesprochen (Lernen mit Kopf, Herz & Hand) |  |  |  |  |  |  |
|  | **Kompetenzorientierung** | Die Bedeutung des Lernstoffs wird den SuS durch die LK deutlich (Gegenwartsbedeutung/Zukunftsbedeutung) |  |  |  |  |  |  |
|  |  | Die SuS beurteilen/reflektieren ihre Leistungen bzw. Kompetenzen selbst |  |  |  |  |  |  |
|  | **Motivierung** | Die Lern- und Leistungsbedarfe der SuS werden berücksichtig, geweckt & erhalten |  |  |  |  |  |  |
|  | **Zielorientierung** | Lernziele werden von der LK klar formuliert & den SuS verdeutlicht |  |  |  |  |  |  |
| **Unterrichtsgestaltung** | **Prozessorientierung** | Die Lernwege/das Lerngeschehen wird von der LK als wichtige Teilkomponente wahrgenommen |  |  |  |  |  |  |
|  | **Selbstständigkeit** | Selbstständiges Lernen wird im Unterricht auf vielfältige Weise gefördert & gefordert |  |  |  |  |  |  |
|  | **Lernerorientierung** | Die LK richtet den Unterricht an den Interessen & Bedürfnissen der SuS aus |  |  |  |  |  |  |
|  | **Partizipation** | Die SuS erhalten Raum den Unterrichts mit zu gestalten |  |  |  |  |  |  |
|  | **Selbstwirksamkeitserleben** | Der Unterricht eröffnet Freiräume für eigene Lern - & Lösungswege |  |  |  |  |  |  |
|  | **Kooperatives Lernen** | Die SuS unterstützen sich gegenseitig beim Lernen / soziale Interaktionen finden statt |  |  |  |  |  |  |
|  | **Methodenvariation** | Die LK nutzt verschiedene Methoden, um den Unterricht zu gestalten |  |  |  |  |  |  |
|  | **Veranschaulichung** | Die Lernprozesse werden durch den Einsatz verschiedener Medien durch die LK gefördert |  |  |  |  |  |  |
|  | **Transparente Leistungserwartungen** | Die LK macht die Anforderungen den SuS transparent /Die SuS wissen zu jeder Zeit, was von ihnen erwartet wird |  |  |  |  |  |  |
|  | **Intelligentes Üben** | Die Übungsphasen passen zum Unterrichtsgeschehen |  |  |  |  |  |  |
|  | **Sicherung** | Gelerntes wird von den SuS ausreichend oft geübt, wiederholt, vertieft & gesichert |  |  |  |  |  |  |
|  | **Differenzierung** | Die LK unterstützt verschiedene Lerntempi, Lernwege & Leistungsniveaus |  |  |  |  |  |  |
|  |  | Die SuS erhalten differenziertes Feedback und Hilfestellungen |  |  |  |  |  |  |
| **Inhalte & Themen** | **Vorwissen aktivieren** | Die Inhalte lassen eine Verknüpfung mit Vorwissen bzw. Vorerfahrungen zu |  |  |  |  |  |  |
|  | **Fächerverbindend** | Es findet ein Transfer zwischen verschiedenen Fächern statt |  |  |  |  |  |  |
|  | **Wirklichkeitsbezug/ Problemorientierung** | Der Unterricht enthält Beispiele aus dem Alltag/ aus der Lebenswelt der SuS/ thematisiert Probleme (authentische Lernerfahrungen) |  |  |  |  |  |  |
| **Klassenführung** | **Strukturiertheit und Klarheit** | Der Unterricht zeigt einen roten Faden (Unterrichtsinhalte bauen aufeinander auf) |  |  |  |  |  |  |
|  |  | Dem Unterrichtsgeschehen liegen erkennbare Regeln zu Grunde |  |  |  |  |  |  |
|  | **Zeitnutzung (Hoher Anteil echter Lernzeit)** | Antworten der SuS bzw. Arbeitsphasen werden von der LK nicht unterbrochen |  |  |  |  |  |  |
|  |  | Die SuS sind fast die gesamte Zeit mit Lernaktivitäten/unterrichtsbezogenen Inhalten beschäftigt |  |  |  |  |  |  |
|  | **Kommunikation** | Die Lautstärke ist der Sozialform & dem Lerninhalt angemessen |  |  |  |  |  |  |
|  |  | Die LK arbeitet mit non-verbaler Kommunikation (Gestik/Mimik/Ritualen)‘ |  |  |  |  |  |  |
|  |  | Arbeitsaufträge, Erklärungen & Impulse werden klar, präzise und kohärent formuliert |  |  |  |  |  |  |
| **konstruktive**  **Unterstützung** | **Lernförderliches**  **Unterrichtsklima** | Die Lernbereitschaft der SuS wird durch die Lehrkraft anerkannt & gefördert |  |  |  |  |  |  |
|  |  | Lernförderlicher Umgang mit Fehlern/ SuS - Beiträge werden als Lernchancen gesehen |  |  |  |  |  |  |
|  |  | Die Lehrkraft ermutigt die SuS Fragen zu stellen/Gedanken zu äußern |  |  |  |  |  |  |
|  |  | Es herrscht eine Atmosphäre von wechselseitiger Wertschätzung & Respekt zw. LK & SuS |  |  |  |  |  |  |
|  |  | …. und zw. SuS & SuS |  |  |  |  |  |  |
| **Lernraum** | **Lernort** | Die Auswahl des Lernorts passt zum Unterrichtsthema |  |  |  |  |  |  |
|  |  | Der Lernorts passt zur Lernphase |  |  |  |  |  |  |
|  |  | Die SuS erschließen den Lernort durch Bewegung & Interaktion |  |  |  |  |  |  |
|  | **Nutzung des Lernorts** | Der Lernort wird aktiv in den Unterricht eingebunden (er wird zum Lerngegenstand) |  |  |  |  |  |  |

**APPENDIX:**

**Anmerkungen (Unterrichtsinhalte/Besonderheiten/Hinweise zum Unterrichtsgeschehen/Sozialformenwechsel):**

Das Beobachtungsprotokoll – OPTIS (Observing Principles of Teaching In School) dient der externen Unterrichtsbeobachtung. Insgesamt ist wichtig, dass mit Hilfe des Beobachtungsprotokolls, OPTIS, keine Bewertung von Unterricht vorgenommen werden soll, sondern Unterricht im Sinne eines ‚Ist-Zustands‘ analysiert bzw. protokolliert wird.

Grundlage für die Erstellung von OPTIS waren Kriterien der Unterrichtsqualität nach: Wiater (1); Charalambous & Praetaerius (2); Praetorius (3); Helmke (4); Kunter & Trautwein (5); KMK (6); Meyer (7); Schilmöller (8); Pieper & Krawinkel (9); Hattie (10); Tulodziecki (11); Muijs & Reynolds (12); Meyer (13); Rickinson et al, (14); Clausen (15); Roßbach (16); Klieme et al., (17); Brophy (18); Deci & Ryan (19); Gerstemmeier & Mandl (20); Berliner (21); Brophy & Allemann (22); Slavin (23).

Es wurden Unterrichtsprinzipien explizit mit Beobachtungskategorien verknüpft, um im Nachfolgenden direkte Ableitungen über das Vorkommenden oder Nicht- Vorkommenden von Unterrichtsprinzipien im beobachteten Unterricht treffen zu können.

Eine 6-stufige Likert Skala dient als Quantifizierungshilfe, um die beobachteten Unterrichtsprinzipien darzustellen. Dabei besteht die Skala aus (- - -) trifft überhaupt nicht zu; (- -) trifft eher nicht zu; (-) trifft selten zu; (+) trifft eher zu, (+ +) trifft häufig zu und (+ + +) trifft vollständig zu.

Im Nachfolgenden sind Beispiele und genaue Sachverhalte für die einzelnen Beobachtungskategorien aufgeführt. Während der Beobachtung sollen nicht die einzelnen Beispiele durchgegangen werden und ‚abgehackt‘ werden. Die aufgeführten Beispiele dienen zur Veranschaulichung und Konkretisierung der zu beobachtenden Kategorien und dienen vor allem als Nachschlagewerk, bzw. als Vorbereitung für bevorstehende Beobachtungen.

Zur Vorbereitung der Beobachtungen müssen die Beobachter geschult werden. Diese Schulung setzt sich aus einer intensiven Auseinandersetzung mit den in der nachfolgenden Tabelle aufgeführten Erläuterungen und Beispielen zusammen (1. Schritt). Zusätzlich sollten frei verfügbare Unterrichtsvideos verwendeten werden, um die Verwendung von OPTIS, sowie das Verständnis über die verschiedenen Beobachtungskategorien zu schärfen (2. Schritt)^[1]^. In einem dritten Schritt sollte mindestens ein ausgefüllter Beobachtungsbogen mit einer weiteren Person diskutiert werden. Insgesamt sollten ca. 8 Stunden für die Schulung mit OPTIS eingeplant werden. Diese Schulung setzt sich aus 3 Teilen zusammen.

Zur Sequenzierung der Beobachtung soll der Sozialformenwechsel genutzt werden. Verwendet die Lehrkraft eine neue Sozialform, soll ein neuer Beobachtungsbogen verwendet werden. Für Sequenzen kürzer als fünf Minuten soll jedoch kein neuer Beobachtungsbogen verwendet werden, da die zu beobachtenden Unterrichtsprinzipien in der Kürze der Zeit schwer beobachtbar sind. In diesem Fall wird der Sozialformenwechsel in den Anmerkungen vermerkt und beide Sozialformen in der Kopfspalte aufgeführt. In diesen Fällen, wird die Länge der jeweiligen stattfindenden Sozialform vermerkt. Dies ist ebenso der Fall, wenn verschiedene Sozialformen simultan stattfinden.

Es wird zwischen 5 verschiedenen Sozialformen unterschieden:

**Frontalunterricht (F),** ist gekennzeichnet durch einen Lehrer- oder aber auch einen SuS-Vortrag. Die restliche Klasse ist passiv und nimmt die Rolle, des/der Zuhörende/n ein.

**Plenum (P),** kennzeichnet sich durch ein Unterrichtsgespräch im gesamten Klassenverbund. Dieses Gespräch kann von der LK, oder von den SuS angeleitet werden.

**Gruppenarbeit (GA),** während dieser Sozialform arbeiten die SuS in Gruppen (größer 2) zusammen. Die LK nimmt eine unterstützende und beratende Funktion ein.

**Partnerarbeit (PA**), ist eine Phase in der die SuS zu zweit Aufgaben bearbeiten und sich mit einander austauschen. Auch hier nimmt die LK eine unterstützende und beratende Rolle ein.

**Einzelarbeit (EA),** ist gekennzeichnet durch alleiniges Arbeiten der SuS. Die LK fungiert auch hier als Unterstützung & Beratung.

Die Beobachtung des Unterrichts soll diesen nicht stören, deshalb sollen sich die BeobachterInnen aus dem Unterrichtsgeschehen rausnehmen und sich eher im Hintergrund aufhalten.

**Erläuterungen zu den Beobachtungskategorien:**

| Unterrichtsprinzipien | Beschreibung/Beispiele der Beobachtungskategorien |
| --- | --- |
| **Handlungsorientierung** | *Inhalte werden von den SuS durch eigenes Handeln erschlossen (dieses Handeln dient als Ausgangspunkt für weiteres unterrichtliches Handeln)*  + der Unterricht ist von der LK entdeckend und individuell konturierend gestaltet  + SuS können durch eigenes Agieren die Inhalte des Unterrichts erleben  + das Handeln der SuS dient als Ausgangspunkt für weiteres unterrichtliches Geschehen  - nur die LK handelt/agiert und die SuS bleiben in der Rolle der passiven ZuhörerInnen bzw. Beobachtenden  - SuS sind passive Empfänger von Instruktionen |
| **Erfahrungsorientierung** | *Die SuS können eigene Erfahrungen im Unterricht sammeln*  + Inhalte des Unterrichts werden durch eigene Erfahrungen auf Seiten der SuS erlebbar gemacht  + die Erfahrungen der SuS im Unterricht regen die SuS zum mitdenken an (Hands & Minds on)  + die SuS führen selbstständig Experimente oder Ähnliches durch  + Erfahrungen der SuS werden im Unterricht weitergenutzt und für spätere unterrichtliche Geschehnisse verwendet  - Experimente oder ähnliche Handlungen werden ausschließlich von der LK durchgeführt  - Inhalte werden lediglich theoretisch vermittelt |
| **Ganzheitlichkeit** | *Verschiedene Sinne der SuS werden im Unterricht angesprochen (Lernen mit Kopf/Herz &Hand)*  + der Unterricht spricht verschiedene Sinne (fühlen, schmecken, riechen, hören, sehen) an  + das sinnliche Handeln unterstützt die Lernprozesse der SuS  + Lernen mit Kopf/Herz und Hand  + der Unterricht löst Emotionen bei den SuS aus (und fördert somit ganzheitliches Lernen)  - der Unterricht spricht nur die kognitiven Reize (Kopf) an  - die Emotionen der SuS haben keinen Raum im Unterricht  - lediglich visuelle und akustische Reizen prägen das Unterrichtsgeschehen |
| **Kompetenzorientierung** | *Die Bedeutung des Lernstoffs wird den SuS durch die LK deutlich (Gegenwartsbedeutung/Zukunftsbedeutung)*  + Bedeutung der Inhalte in einem globalen Verständnis von Bildung/Lernen wird deutlich (Kompetenzen werden erlernt/ weg von der reinen Inhaltsorientierung von Unterricht)  - die LK fokussiert nur die Vermittlung von Inhalten (der Prozess hat dabei keine Bedeutung)  - SuS tun (schreiben, lesen…) nur um eine Aufgabe zu erfüllen, ohne zu verstehen warum und wofür |
|  | *Die SuS beurteilen/reflektieren ihre Leistungen bzw. Kompetenzen selbst*  + Wichtigkeit der Reflexion des eigenen Lernens (metakognitive Kompetenzen, Selbstmonitoring)  + Thematisierung von Methoden der optimalen Vor- und Nachbereitung von Gelerntem  + die LK unterstützt die SuS im Aufbau von Lernstrategien und diese versuchen diese anzuwenden  + Thematisierung der Wichtigkeit von Lern- und Gedächtnisstrategien, Lerntechniken, Zeitplanung  + Strategien, wie man z. B. die Hausaufgaben erledigt, einen Wochenplan erstellt, Stationen durchläuft; werden gemeinsam besprochen und reflektiert  + Hinweise auf bereits eingeführte Lernstrategien, z.B. auf Nachschlagewerke, mögliche Hilfsmittel, Suchmaschinen wie Google  - Leistungen werden nur an Hand von Noten (Output) durch die LK festgemacht  - nur die LK fungiert als Kontrollorgan  - die SuS werden nicht dazu angeregt, die eigenen Leistungen selbst einzuschätzen |
| ***Motivierung*** | *Die Lern- und Leistungsbedarfe der SuS werden berücksichtig, geweckt & erhalten (Neugier)*  + der Unterricht weckt die Neugier der SuS  + Lernaufgaben werden von der LK so formuliert und begleitet, dass sie die SuS zum Denken & Lernen motiviert werden  + die LK motiviert die SuS am Unterricht teilzuhaben (Begeisterungsfähigkeit)  + der Unterricht weckt, erhält und berücksichtigt die Lernbedürfnisse der SuS  + der Unterricht weckt, erhält und berücksichtigt die Leistungsbedürfnisse der SuS  + die LK nimmt Zeichen des Unmotiviert-seins wahr und ergründet die Ursachen  + SuS arbeiten mit, melden sich, sind aktiv („ontask“-Verhalten)  + SuS engagieren sich in Gruppenarbeit, Partnerarbeit, Einzelarbeit  + der Unterricht fordert, aber über- oder unterfordert die SuS nicht  - SuS sind erkennbar lustlos, passiv, gähnen, gehen alternativen Tätigkeiten nach ("offtask"-Verhalten)  - SuS sind vom Unterricht gelangweilt oder überfordert  - die LK nimmt Zeichen des Unmotiviert-seins/"offtask"-Verhalten wahr, aber ignoriert diese  - der Unterricht unter- oder überfordert die SuS |
| **Zielorientierung** | *Lernziele werden von der LK klar formuliert und den SuS verdeutlicht*  + die Lernziele des Unterrichts werden von der LK explizit angesprochen (muss nicht unbedingt zum Stundenbeginn sein)  + die LK diskutiert mit den SuS gemeinsam, warum die Inhalte/Themen im Unterricht behandelt werden  + die LK visualisiert die Ziele der Stunde/der Unterrichtseinheit  + Unterrichtsziel werden im Unterrichtsverlauf von der LK rekapituliert/aufgegriffen  + SuS formulieren selbst was sie lernen wollen/sollen (individuelle Zielformulierung)  + zu Unterrichtsende wird eine Verbindung von Gelerntem und den Lernzielen aufgezeigt (oder von den SuS hergestellt)  + die LK prüft und diskutiert mit den SuS, ob die Ziele am Ende des Unterrichtsgeschehens erreicht wurden  **-** SuS wissen nicht, warum sie gerade unterrichtete Inhalte erlernen/verstehen sollen  - Thematisierung der Wichtigkeit von Lernen für Prüfungen bzw. Noten |
| **Prozessorientierung** | *Die Lernwege/das Lerngeschehen wird von der LK als wichtige Teilkomponente wahrgenommen*  + SuS wird verdeutlicht, dass Lernen ein Prozess ist und dass jeder Teilschritt entscheidend ist  + Zwischenprodukte werden diskutiert und Bedürfnisse der SuS für nachfolgende Schritte besprochen  + auch Teilleistungen haben eine Relevanz und werden wertgeschätzt  - die LK fokussiert auf Lernprodukte (Tests/Klausuren/Hausaufgaben), die erstellt und abgegeben werden müssen  - die LK thematisiert nur Endprodukte (‚mach das erstmal fertig‘ – und es finden keine Zwischenevaluationen statt |
| **Selbstständigkeit** | *Selbstständiges Lernen wird im Unterricht auf vielfältige Weise gefördert & gefordert*  + die LK übernimmt eine beobachtende/lern-begleitende/moderierende Rolle  + die SuS wählen sich selbstständig Lernpartner/innen  + die SuS übernehmen Verantwortung für ihren Lernprozess  + die SuS reflektieren ihren eigenen Lernprozess (und leiten daraus Maßnahmen ab)  - die LK trifft die Entscheidungen für die SuS  - die SuS warten darauf, von der LK aufgefordert zu werden, um etwas zu tun |
| ***Lernerorientierung*** | *Die LK richtet den Unterricht an den Interessen & Bedürfnissen der SuS aus*  + SuS dürfen Gesprächsleitende Funktionen, auf inhaltlicher oder organisatorischer Ebene übernehmen (wenn sie wollen)  + von den SuS selbst mitgebrachte Gegenstände und Rechercheergebnisse werden in den Unterricht mit einbezogen  + die LK kennt die Interessen der SuS und schafft entsprechende Lernangebote  + die LK fragt nach den Interessen der SuS und interessiert sich für die SuS  + die LK reagiert flexibel auf unterrichtsbezogene Bedürfnisse der SuS und kann den Unterricht auch spontan daran ausrichten  + die Lk baut kurze (spontane) Bewegungs- oder Interaktionsphase ein, wenn sie merkt, dass die SuS sich nicht mehr konzentrieren können (nimmt das Bedürfnis auf Unterbrechung wahr)  - von den SuS verbalisierte Bedürfnisse werden von der LK negiert bzw. als unwichtig gewertet  - die LK arbeitet ausschließlich den Lehrplan/das Lehrmaterial ab  - die LK kann auch spontane Bedürfnisse der SuS nicht eingehen und hält an dem geplanten Unterrichtsvorgehen fest  - die Bedürfnisse der SuS werden von der LK nicht berücksichtigt |
| ***Partizipation*** | *Die SuS erhalten Raum den Unterricht mit zu gestalten*  + die SuS werden in die Auswahl der Unterrichtsthemen mit einbezogen  + die SuS sind in organisatorische Aspekte des Unterrichts involviert  + die SuS übernehmen Verantwortung für den Unterricht  + die LK geht auf Impulse, Ideen und Initiativen der SuS ein und greift diese auf  + die LK schafft einen Rahmen/Raum, in dem die SuS Entscheidungen treffen können  - die LK entscheidet alleine über Inhalte des Unterrichts  - die LK unterbindet Wünsche, Ideen und Initiativen der SuS zur Partizipation |
| **Selbstwirksamkeitserleben** | *Der Unterricht eröffnet Freiräume für eigene Lern- & Lösungswege*  + die LK gibt Hilfen (Kontrollzettel) zur Selbstkontrolle (Beispiele der Kontrolle: Partner-, Selbstkontrolle, …)  + SuS vergleichen Lösungswege miteinander  + SuS beurteilen sich und ihre Leistungen selbst  + SuS beschreiben sich gegenseitig den Lernprozess  + SuS reflektieren den Lernprozess, z.B. durch ‘was habe ich bis jetzt erreicht? Wo will ich hin?  + SuS entscheiden, selbstständig, an was sie gerade arbeiten wollen  + SuS erklären sich gegenseitig bestimmte Unterrichtsinhalte (z.B. Lernen durch Lehren, Lernpartnerschaften)  + SuS planen Lernprozesse, Unterrichtseinheiten und deren Umsetzung selbstständig und eigenverantwortlich (z.B. Informationen sammeln und auswerten, Methoden einsetzen und Medien nutzen)  + SuS präsentieren ihre Ergebnisse und diskutieren diese im Plenum  + SuS formulieren, was sie lernen sollen, woran sie arbeiten  + SuS formulieren sich selbst Ziele und überprüfen dessen Erreichung (Möglichkeit und Chancen der Selbstbewertung)  + (dadurch wird) eine Selbstkompetenz der SuS aufgebaut  + Selbstvertrauen, Selbstkonzept, Selbstwertgefühl, Selbstwirksamkeit werden als wichtige Teilaspekte des Lernens angesehen  + Die LK unterstützt das Selbstvertrauen (usw.) der SuS  - die LK ist der Entscheider/Allmächtigkeits-Rolle  - die LK ist so dominant, dass SuS keinen Raum haben  - nur die LK entscheidet, was gut war und was nicht  - SuS müssen die gleichen Lösungsweg gehen, auch wenn sie Ideen für einen eigenen haben |
| **Kooperatives Lernen** | *Die SuS unterstützen sich gegenseitig beim Lernen / soziale Interaktionen finden* *statt*  + Lernen findet (auch) in Gruppen statt  + SuS unterstützen sich bei der Bearbeitung von Aufgaben  + Aufforderung zu oder Thematisierung (Wichtigkeit, Nützlichkeit) von Hilfsbereitschaft/ Teamfähigkeit, Höflichkeit, Respekt, Mitleid, Empathie, Perspektiven- und Rollenübernahme, Toleranz  + Thematisierung von Klassenrat, Streitschlichtung  + Hinweise zum Umgang miteinander, Regeln der Kommunikation, z.B. Ausreden lassen, niemanden auslachen, Gesprächs-, Klassen- und andere Regeln einhalten, freundlich zueinander sein, Umgangston beachten  + Thematisierung der Integration unterschiedlicher Schüler/innen (z.B. Integration beeinträchtigter Kinder)  + SuS werden auch untereinander als wertvolle Lernbegleiter und Helfer angesehen  - SuS arbeiten ausschließlich in Einzelarbeit  - SuS erhalten keinen Raum, sich mit MitschülerInnen über Inhalte und Erfahrungen auszutauschen  - soziales Lernen wird von der LK aus Zeitfresser wahrgenommen  - nur die LK wird als HelferIn angesehen (gegenseitiges Helfen der SuS findet nicht statt/wird unterbunden) |
| **Methodenvariation** | *Die LK nutzt verschiedene Methoden, um den Unterricht zu gestalten*  + die LK nutzt verschiedene Methoden (z.B. Gruppenpuzzel, Tandem, Experten…)  + die LK nutzt verschiedene Lehr-Lern-Formen  - die LK unterrichtet z.B. hauptsächlich im Frontalunterricht (keine Methodenvariation)  - Methoden werden nicht variiert – die LK nutzt die gleichen Methoden für verschiedene Inhalte immer wieder  - die LK versucht möglichst viele Methoden in den Unterricht zu integrieren, so dass die Unterrichtsinhalte/Abläufe darunter leiden (Quantität vor Qualität) |
| **Veranschaulichung** | *Die Lernprozesse werden durch den Einsatz verschiedener Medien durch die LK gefördert*  + die LK nutzt verschiedene Medien (Bücher, Arbeitsblätter, Tablets, Präsentationen, Smartboards ...) um den Unterricht zu gestalten  + die LK nutzt Visualisierungen (Bilder, Videos und andere Medien) um die sinnliche Wahrnehmung zu unterstützen  + Erklärungs- bzw. Lernprozesse werden durch unterschiedliche Medien unterstützt  + Veranschaulichungen (mit Hilfe von Medien/Materialen) werden von der LK als Erkenntnis- und Verstehenshilfen eingesetzt  + komplizierte Sachverhalte werden mit Hilfe von Medieneinsatz verständlicher dargestellt-  - verschiedene Medien werden im Unterricht nicht genutzt  - Medien werden Zusammenhangslos eingesetzt |
| **Transparente Leistungserwartungen** | *Die LK macht die Anforderungen den SuS transparent /Die SuS wissen zu jeder Zeit, was von ihnen erwartet wird*  + die LK legt zu Beginn des Unterrichts die Anforderungen transparent dar  + die Erwartungen an die SuS sind transparent (und werden von der LK kommuniziert)  + SuS wissen, was von ihnen erwartet wird  + die LK begleitet den Lernweg der SuS  + SuS erhalten zu einzelnen Zwischenschritten eine Rückmeldung  + Teilschritte/Zwischenergebnisse werden gemeinsam besprochen und weiteres Vorgehen diskutiert  - die LK gibt ausschließlich am Ende eines Lernprozesses Rückmeldung  - die Leistungsanforderungen tauchen irgendwann plötzlich auf  - die SuS wissen nicht, was von Ihnen erwartet wird |
| **intelligentes Üben** | *Die Übungsphasen passen zum Unterrichtsgeschehen*  + Übungsphase werden von den SuS als sinnstiftend empfunden und genutzt  + das Feedback der LK ist konstruktiv und individuell und hilft dem SuS zu verstehen, wie er/sie weiter vorgehen kann  + SuS haben verstanden, was sie üben sollen und erhalten dafür ausreichend Zeit  + Geübtes (z.B. Hausaufgaben) werden von der LK anerkannt und gewürdigt  + während Übungsphasen ist die LK aufmerksam und ansprechbar  - während Übungsphasen muss die LK die SuS immer wieder zum ‚weitermachen‘ auffordern  - SuS wissen nicht, was sie gerade Üben sollen |
| **Sicherung** | *Gelerntes wird von den SuS ausreichend oft geübt, wiederholt, vertieft & gesichert*  + Unterrichtsinhalte bauen aufeinander auf und vorangegangen Inhalte werden gefestigt  + Unterrichtsinhalte werden immer wieder aufgegriffen und angewendet (Wissenskonstruktion)  + verschiedene Sicherungsstrategien werden durch die LK angeleitet und von den SuS angewendet (kognitiv wird verknüpft mit anderen Sinnen)  + SuS üben und vertiefen Gelerntes, um es später abrufen zu können  + es werden Verknüpfungen mit Gelerntem hergestellt  - Inhalte bleiben isoliert stehen und werden weder wiederholte und verknüpft  - Hausaufgaben (welche zur Sicherung dienen) werden nicht wertgeschätzt bzw. thematisiert |
| **Differenzierung** | *Die LK unterstützt verschiedene Lerntempi, Lernwege & Leistungsniveaus (evtl. durch differenzierte Aufgabenstellungen)*  + die LK bietet den SuS differenzierte Ausgabenstellungen an  + SuS können je nach eigenem Können, individuelle Lernwege bestreiten  + die verschiedenen Fähigkeiten der SuS werden berücksichtig und im Unterricht genutzt  + verschiedene SuS werden je nach Bedürfnissen, unterschiedliche Zeitvorgaben oder Aufgabenstellungen gegeben  + SuS erhalten Wahlmöglichkeiten (in Abhängigkeit vom eigenen Können)  + die LK hat unterschiedliches Material für verschiedenen SuS vorbereitet  - die LK gibt eine einheitliche, für alle SuS verbindliche Taktung des Unterrichts vor  - alle SuS müssen die gleichen Lernwege bestreiten (die LK unterbinden eigene Lernwege)  - Weitergabe von Aufgaben in die Hausaufgabe (Quasi-Strafarbeit für langsame SuS)  - alle SuS erhalten die gleichen Aufgabenstellungen/Materialien, keine Wahlmöglichkeit |
|  | *Die SuS erhalten differenziertes Feedback und Hilfestellungen*  + die LK geht in der Klasse herum und unterstützt individuell die SuS  + die LK unterstützt und berät einzelne SuS  + die LK ermuntert die SuS in ihrem eigenen Lernfortschritt und sieht jeden Lernfortschritt als wertvoll an  + die LK erklärt einzelnen SuS nochmals den Unterrichtsinhalt/die Aufgaben  - die LK wertet individuelle Einzelleistungen ab – nur die zu erreichende Norm ist lobenswert  - SuS erhalten negative Rückmeldungen, weil sie ‚zu langsam‘ waren  - das Feedback der LK ist nicht Zukunftsorientiert/ undifferenziert (‚das war gut‘) |
| **Vorwissen aktivieren** | *Die Inhalte lassen eine Verknüpfung mit Vorwissen bzw. Vorerfahrungen zu (Wissens/Inhaltsebene)*  + die LK greift auf Wissen/Erfahrungen aus dem Alltag zurück und stellt Zusammenhänge her  + die LK greift bereits Gelerntes explizit auf  + die LK stellt aktiv Zusammenhang von Neuem und bereits Gelerntem her  + die LK schafft Verknüpfungen z.B. von Hausaufgaben mit neuem Lernstoff  - Vorwissen bzw. Vorerfahrungen werden nicht aktiviert  - Zusammenhänge werden den SuS nicht explizit aufgezeigt |
| **Fächerverbindend** | *Es findet ein Transfer zwischen verschiedenen Fächern statt*  + die LK stellt einen Transfer von Unterrichtsinhalten zu anderen Unterrichtsfächern her  + Beispiele aus anderen Unterrichtsfächern werden zum Erklären verwendet  - Unterrichtsinhalte bleiben isoliert von anderen Themenbereichen und anderen Fächern  - verknüpfendes Denken wird von der LK nicht gefördert |
| **Wirklichkeitsbezug/ Problemorientierung** | *Der Unterricht enthält Beispiele aus dem Alltag/ aus der Lebenswelt der SuS/ thematisiert Probleme (authentische Lernerfahrungen)*  + die LK arbeitet mit Beispiele/Aufgaben, welche an den Erfahrungskontext, Interessen, außerschulischen Aktivitäten, Lebenshorizont der SuS anknüpfen  + der Unterricht regt durch die Wahl der Inhalte die SuS zum Lernen an (intrinsische Motivation wird durch Lernbegeisterung sichtbar)  + die LK thematisiert, dass und warum der Unterrichtsstoff auch für den Alltag relevant ist  + Unterricht hat einen aktuellen Bezug (Anknüpfung an Print- oder Pressemeldung, Internetmeldung oder -forum)  + die LK geht auf Alltagswissen der SuS ein  + die LK bereitet Unterricht mit SuS-Bezug vor  + die LK geht auf die Lebenswelt der SuS ein (z. B durch Beispiele)  + Mehrdeutigkeiten, Zweifel und Wiedersprüche werden im Sinne des problemlösenden Lernens diskutiert  - Beispiele werden aus dem Lehrbuch verwendet, ohne Bezug zu der Lebenswirklichkeit der SuS – veraltetes Material  - Pseudo-Beispiele werden verwendet (ohne wirklichen SuS-Bezug) |
| **Strukturiertheit und Klarheit** | *Der Unterricht zeigt einen roten Faden (Unterrichtsinhalte bauen aufeinander auf)*  + zu Beginn der Stunde wird dargestellt was passieren wird  + es gibt innerhalb einer Unterrichtsphase Rückbezüge auf Vorheriges  + Inhalte innerhalb einer Unterrichtsstunde/Unterrichtseinheit bauen aufeinander auf  + die formulierten Lernziele werden von der LK im Unterrichtsverlauf wieder aufgegriffen  - die LK stellt keinen Zusammenhang zwischen den verschiedenen Unterrichtsphasen her  - die LK spring von einem Thema zum anderen (kein roter Faden)  - Dysfunktionales Abschweifen, Verzetteln |
|  | *Dem Unterrichtsgeschehen liegen erkennbare Regeln zu Grunde*  + es gibt klare Regeln an die sich alle halten (SuS und LK)  + den Interaktionen im Klassenzimmer liegt ein erkennbares Regelsystem zu Grunde  + gemeinsam aufgestellte Regeln sind im Klassenzimmer festgehalten und bei Bedarf kann die LK auf diese verweisen  + wenn notwendig, greift die LK auf existierende Regeln zurück und führt diese den SuS vor Augen  + SuS weisen sich gegenseitig auf gemeinsam aufgestellte Regeln hin  - es herrscht ein Durcheinander & Chaos in der Klasse, dass den Unterricht stört  - die LK muss durch verbale Lautstärke oder ‚Bestrafungen‘ Ordnung in der Klasse herstellen |
| **Zeitnutzung (hoher Anteil echter Lernzeit)** | *Antworten der SuS bzw. Arbeitsphasen werden von der LK nicht unterbrochen*  + SuS erhalten genügend Zeit, sich auf Fragen eine Antwort zu überlegen (> 3 Sekunden)  + die LK tritt in Arbeitsphase aktive in den Hintergrund und lässt die SuS arbeiten  + die LK macht klare Zeitangabe für Aufgabenphasen (evtl. durch Visualisierung oder verbal)  + SuS wissen wie lange sie für Aufgaben/ bestimmte Unterrichtsphase Zeit haben  + die LK erinnert zwischendurch an die Zeit (oder Zeitwächter-Aufgaben)  - Arbeitsphasen werden plötzlich durch die LK unterbrochen  - die LK gibt die (unbeantwortete) Frage sofort an andere SuS weiter  - die LK beantwortet, an die SuS gestellte Frage selbst  - nur die „schnellen“ SuS kommen zu Wort |
|  | *Die SuS sind fast die gesamte Zeit mit Lernaktivitäten/unterrichtsbezogenen Inhalten beschäftigt*  + Pünktlichkeit der LK und der SuS (somit pünktlicher Start des Unterrichts)  + Material und Geräte liegen bereit  + zügiger Wechsel zwischen Unterrichtsphasen, z.B. bei Änderung der Sozialform (durch Rituale; eingeübte Vorgänge)  + Auslagerung von „Administrativa“ und „Orgakram“  + Die SuS sind aktiv am Unterrichtsgeschehen beteiligt  + intensive Lernphase und erholsame Pausen wechseln sich ab  + die LK kann mit unvorhergesehenen Störungen umgehen, sodass diese den Unterrichtsverlauf nicht nachhaltig beeinträchtigen/stören  - schleppender, zögerlicher Wechsel, z.B. bei Änderung der Sozialform  - Zeitverlust durch Probleme mit der Technik  - SuS warten auf Kontrolle der erledigten Aufgaben oder auf neue Arbeitsaufträge (unnötige Wartezeit)  - Unterricht endet vorzeitig  - die SuS sind abgelenkt und nicht bei der Sache  - unvorhergesehene Störungen führen zu andauernden/nachhaltigen Beeinträchtigungen des Unterrichts |
| **Kommunikation** | *Die Lautstärke ist der Sozialform & dem Lerninhalt angemessen*  + SuS bekommen das Ende/den Anfang von Unterrichtsphasen mit  + Aufgabenstellungen/Redebeiträge können von allen gehört werden  + in Partner- oder Gruppenarbeitsphase toleriert die LK eine erhöhte Arbeitslaufstärke (und drängt nicht auf Ruhe)  - lauter werden der LK (oder anderer SuS) um die Klasse zu „übertrumpfen“  - Die LK stört den Unterricht durch unnötiges Handeln |
|  | *Die LK arbeitet mit non-verbaler Kommunikation (Gestik/Mimik/Ritualen)‘*  + die LK nutzt non-verbale Kommunikation, um die verbale Kommunikation zu unterstreichen  + der Unterricht wird durch Rituale und non-verbale Kommunikation von der LK strukturiert  + die LK nutzt non-verbale Kommunikation um die Aufmerksamkeit der SuS zugewinnen  + eingeübte Rituale geben dem Unterricht und den SuS Struktur  - die einzige Form der Kommunikation ist die Stimme/Sprache |
|  | *Arbeitsaufträge, Erklärungen & Impulse werden klar, präzise und kohärent formuliert*  + die LK erklärt Arbeitsaufträge klare, unmissverständliche und präzise  + die LK verwendet eine angemessene Aussprache, Rhetorik und Diktion  + die Geschwindigkeit und Lautstärke von Arbeitsaufträgen ist verständlich & passt zum Unterrichtsgeschehen  - die LK äußert sich in Ein-Wort-Sätzen oder unvollständigen Sätzen  - das Auftreten der LK ist von Marotten, Manierismen, Sprechverzögerungen („ähmmm“) geprägt  - die LK nutzt viele Unsicherheits- und Vagheitsausdrücke („sozusagen“, „irgendwie“, „sag-ich-mal“)  - die LK spricht mit mangelnder und unsichere Grammatik und Wortschatz |
| **Lernförderliches Unterrichtsklima** | *Die Lernbereitschaft der SuS wird durch die Lehrkraft anerkannt und gefördert*  + die LK unterstützt die Lernaktivität der SuS durch verbale Äußerungen/Gestik/Mimik  + die LK stellt weiterführende Fragen/ gibt Hinweise (um die Lernaktivität der SuS weiter zu fördern)  + die LK fördert die Lernbereitschaft der SuS bzw. erkennt diese an  + sollten SuS nicht weiterkommen, bietet die LK Hilfe an (Prozessbegleitung)  - LK ignoriert die intrinsische Lernmotivation der SuS (sieht diese als selbstverständlich an) |
|  | *Lernförderlicher Umgang mit Fehlern/ SuS - Beiträge werden als Lernchancen gesehen*  + die LK verdeutlicht, dass Fehler wichtig sind (als Lernchancen angesehen werden)  + SuS erfahren nicht nur, DASS etwas falsch ist, sondern WARUM  + die LK gibt Hinweise, die das Verständnis fördern  + die LK lässt die SuS ihre Fehler selbst korrigieren  + die LK bindet SuS in Fehlerbearbeitung mit ein  + Fehler werden als Lernchancen gesehen, um noch nicht Verstandenes zu identifizieren und aufzugreifen  + die LK berichtigt Fehler nicht nur, sondern ergründet die Ursache (greift nicht Verstandenes auf)  - SuS-Fehler werden durch die LK ignoriert bzw. fehlerhafte Äußerungen abgewiegelt  - SuS werden bei Fehlern vorgeführt/ ihr ‚Versagen‘ wird dargestellt  - Fehler werden von der LK aus „schlecht“ angesehen |
|  | *Die Lehrkraft ermutigt die SuS Fragen zu stellen/ Gedanken zu äußern*  + Fragen werden als Lernchancen gesehen, um noch nicht Verstandenes zu identifizieren und aufzugreifen  + die LK ermutigt SuS Fragen zu stellen (durch verbale Aufforderungen)  + Zeit für Fragen wird von der LK bei der Planung des Unterrichts mit eingeplant  - die LK unterdrückt Fragen  - die LK bricht Fragen ab („dass musst du nun aber wirklich verstanden haben…“)  - der Raum für Fragen muss am Ende entfallen, weil keine Zeit mehr ist |
|  | *Es herrscht eine Atmosphäre von wechselseitiger Wertschätzung und Respekt zw. LK & SuS*  + Der wechselseitige Umgangston ist durch Höflichkeit und Respekt gekennzeichnet, sowohl von der LK, als auch von den SuS  + Ausdrückliche Begrüßung und Verabschiedung  + freundliche Anrede  + die LK geht auf persönliche und private Belange von SuS ein (nimmt Schüler als Persönlichkeit wahr, nicht nur als zu Belehrende)  + Verbaler und nonverbaler Ausdruck von Sympathie und Herzlichkeit  + die LK nimmt SuS-Vorschläge ernst  + es herrscht eine Angstfreie Atmosphäre  - Äußerungen, die auf mangelnde Wertschätzung schließen lassen: Ironie, Spott, Verächtlichmachung, Fluchen  - Äußerungen oder nonverbaler Ausdruck von geringer Wertschätzung  - die LK droht (z.B. mit Leistungsüberprüfungen, schlechten Noten, …)  - es herrscht ein stark hierarchisches Gefälle zw. LK und SuS, dass von der Dominanz der LK geprägt ist |
|  | …. *und zw. SuS & SuS*  + SuS lassen sich gegenseitig ausreden, Wertschätzen Beiträge der Anderen (respektvoller Umgang)  + SuS nehmen die anderen SuS als wertvolle Austauschpartner wahr  + Redebeiträge andere SuS werden konzentriert aufgenommen  + SuS helfen sich gegenseitig (Hilfsbereitschaft)  - SuS unterbrechen sie gegenseitig  - einzelne SuS werden ausgeschlossen  - SuS nehmen nur die LK aus wertvolle Unterstützung wahr |
| **Lernort** | *Die Auswahl des Lernorts passt zum Unterrichtsthema*  + Lerninhalte und Lernort ergänzen sich  + der Lernort eröffnet Raum für handlungs- und erfahrungsorientiertes Lernen  + der Lernort wird von der LK bewusst gewählt, um das Unterrichtsthema/Lernprozesse zu unterstützen  + der Lernort kann Beispiele (Wirklichkeitsbezug) für den Unterricht bieten  - der Lernort weißt keinen Zusammenhang mit unterrichtlichen Inhalten auf (keine bewusste, zielgeleitete Entscheidung für den Lernort – Willkürlichkeit des Lernorts) |
|  | *Der Lernort passt zur Lernphase*  + die Lernumgebung ermöglicht ein vertieftes Verständnis des Lernstoffes  + der Lernort wird von der LK bewusst gewählt, um das Lernen (in der entsprechenden Lernphase) zu unterstützen  + der Lernort unterstützt die jeweilige Lernphase (z.B. durch entdeckendes Lernen zum Erschließen von neuen Unterrichtsthemen)  - der Lernort durch z.B lauten Straßenverkehr oder viele ablenkende Personen stört Lernphasen, die eher aus Konzentration oder Wahrnehmung abzielen  - der Lernort stört (z.B durch Ablenkungen) die Lernphase |
|  | *Die SuS erschließen den Lernort durch Bewegung und Interaktion*  + SuS bewegen sich im Raum und erschließen somit den Lernort durch eigenes Handeln  + Lernen und Bewegung wird durch den Lernort gefördert  + SuS treten mit der Lernumgebung in Interaktion (entweder mit dem Raum oder den Menschen in dem Raum)  - SuS sitzen an einem Ort und bewegen sich während der Lernphase nicht  - es findet keine Interaktion zwischen den SuS und dem Lernort statt |
| **Nutzung des Lernorts** | *Der Lernort wird aktiv in den Unterricht eingebunden*  + Die LK nutzt Gegenstände oder die Umgebung um den Unterricht mit Beispielen zu veranschaulichen  + der Lernort selbst wird zum Lerngegenstand  - Unterricht findet wie im Klassenzimmer statt; „nur“ halt an einem anderen Ort |

* die Appendix von Andreas Helmke (2012)- ELL dient als Orientierung

**References**

1. Wiater W. Unterrichtsprinzipien: Prüfungswissen - Basiswissen Schulpädagogik. 8th ed. Donauwörth: Auer; 2021.

2. Charalambous CY, Praetorius A-K. Creating a forum for researching teaching and its quality more synergistically. Studies in Educational Evaluation. 2020;67:100894.

3. Praetorius A-K, Klieme E, Herbert B, Pinger P. Generic dimensions of teaching quality: the German framework of Three Basic Dimensions. ZDM – Mathematics Education. 2018;50(3):407–26.

4. Helmke A. Unterrichtsqualität und Lehrerprofessionalität. Diagnose, Evaluation und Verbesserung des Unterrichts. 5th ed. Stuttgart: Klett; 2014.

5. Kunter M, Trautwein U. Psychologie des Unterrichts. Paderborn: Ferdinand Schöningh; 2013.

6. Kultus Minister Konferenz (KMK). Konzeption der Kultusministerkonferenz zur Nutzung der Bildungsstandards für die Unterrichtsentwicklung. KMK: 2010.

7. Meyer H. Qualitätsmerkmale guten Unterrichts in der Diskussion. In: Fischer C, Schilmöller R, editors. Was ist guter Unterricht? Qualitätskriterien auf dem Prüfstand. Münster: Aschendorff; 2010. p. 6–38.

8. Schilmöller R. Guter Unterricht - Gute Schule. Notwendige Kontexte gelingender Unterrichtspraxis. In: Schilmöller R, Fischer C, editors. Was ist guter Unterricht? Qualitätskriterien auf dem Prüfstand. Münster: Aschendorff; 2010.

9. Pieper U, Krawinkel U. Handlungsorientiertes Lernen an der Albert-Schweitzer-Schule Münster. In: Fischer C, Schilmöller R, editors. Was ist guter Unterricht? Qualitätskriterien auf dem Prüfstand. Münster: Aschendorff; 2010. p. 169–75.

10. Hattie J. Visible learning: A synthesis of meta-analyses relating to achievement. London [u.a.]: Routledge; 2009.

11. Tulodziecki G, Herzig B, Blömeke S. Gestaltung von Unterricht. Eine Einführung in die Didaktik. Bad Heilbrunn: Klinkhardt.

12. Muijs D, Reynolds D. Effective Teaching. Evidence and Practice. London: Sage; 2005.

13. Meyer H. Zehn Merkmale guten Unterrichts. Empirische Befunde und didaktische Ratschläge. Pädagogik. 2003;10:36–43.

14. Rickinson M, Dillon J, Teamey K, Morris M, Choi M, Sanders K, and Benefield P. A review of research on outdoor learning. 2004.

15. Clausen M. Unterrichtsqualität: Eine Frage der Perspektive? Münster: Waxmann; 2002.

16. Roßbach H-G. Unterrichtsqualität im 2. Schuljahr - Ergebnisse einer empirischen Untersuchung. Unterrichtswissenschaft. 2002;30:230–45.

17. Klieme E, Schümer G, Knoll S. Mathematikunterricht in der Sekundarstufe I: Aufgabenkultur und Unterrichtsgestaltung. In: BMBF, editor. TIMSS - Impulse für Schule und Unterricht, Forschungsbefunde, Reforminitiativen, Praxisberichte und Video-Dokumente. Bonn: BMBF; 2001. p. 43–58.

18. Brophy J. Teaching. Educational Practices Series-1. 2000.

19. Deci EL, Ryan RM. The “What” and “Why” of Goal Pursuits: Human Needs and the Self-Determination of Behavior. Psychological Inquiry. 2000;11:227–68.

20. Gerstenmaier J, Mandl H. Wissenserwerb unter konstruktivistischer Perspektive. Zeitschrift für Pädagogik. 1995;41:867–88.

21. Berliner DC. Telling the stories of educational psychology. Educational Psychologist. 1992;27:143–61.

22. Brophy J, Alleman J. Activities as instructional tools: A framework for analysis and evaluation. Educational researcher. 1991;20(4):9–23.

23. Slavin RE. Cooperative Learning: Theory, Research, and Practice: Prentice Hall; 1990.

1. Eine Vielzahl an frei zugänglichen Unterrichtsvideos ist auf der Internetseite : <https://www.fdz-bildung.de/videoportale> verfügbar.
